# A proteomic signature that reflects pancreatic beta-cell function

**DOI:** 10.1371/journal.pone.0202727

**Published:** 2018-08-30

**Authors:** Aoife M. Curran, Marie Pier Scott-Boyer, Jim Kaput, Miriam F. Ryan, Elaine Drummond, Eileen R. Gibney, Michael J. Gibney, Helen M. Roche, Lorraine Brennan

**Affiliations:** 1 Institute of Food and Health, UCD School of Agriculture and Food Science, University College Dublin, Belfield, Ireland University College Dublin, Dublin, Republic of Ireland; 2 Food for Health Ireland (FHI), University College Dublin, Belfield, Ireland University College Dublin, Dublin, Republic of Ireland; 3 The Microsoft Research – University of Trento Centre for Computational and Systems Biology (COSBI), Rovereto, Italy; 4 Nestlé Institute of Health Sciences, Lausanne, Switzerland; 5 Nutrigenomics Research Group, UCD Conway Institute of Biomolecular and Biomedical Research and UCD Institute of Food and Health, School of Public Health, Physiotherapy and Sports Science, University College Dublin, Belfield, Dublin, Republic of Ireland; University of Michigan, UNITED STATES

## Abstract

**Aim:**

Proteomics has the potential to enhance early identification of beta-cell dysfunction, in conjunction with monitoring the various stages of type 2 diabetes onset. The most routine method of assessing pancreatic beta-cell function is an oral glucose tolerance test, however this method is time consuming and carries a participant burden. The objectives of this research were to identify protein signatures and pathways related to pancreatic beta-cell function in fasting blood samples.

**Methods:**

Beta-cell function measures were calculated for MECHE study participants who completed an oral glucose tolerance test and had proteomic data (n = 100). Information on 1,129 protein levels was obtained using the SOMAscan assay. Receiver operating characteristic curves were used to assess discriminatory ability of proteins of interest. Subsequent *in vitro* experiments were performed using the BRIN-BD11 pancreatic beta-cell line. Replication of findings were achieved in a second human cohort where possible.

**Results:**

Twenty-two proteins measured by aptamer technology were significantly associated with beta-cell function/HOMA-IR while 17 proteins were significantly associated with the disposition index (p ≤ 0.01). Receiver operator characteristic curves determined the protein panels to have excellent discrimination between low and high beta-cell function. Linear regression analysis determined that beta-endorphin and IL-17F have strong associations with beta-cell function/HOMA-IR, β = 0.039 (p = 0.005) and β = -0.027 (p = 0.013) respectively. Calcineurin and CRTAM were strongly associated with the disposition index (β = 0.005 and β = 0.005 respectively, p = 0.012). *In vitro* experiments confirmed that IL-17F modulated insulin secretion in the BRIN-BD11 cell line, with the lower concentration of 10 ng/mL significantly increasing glucose stimulated insulin secretion (p = 0.043).

**Conclusions:**

Early detection of compromised beta-cell function could allow for implementation of nutritional and lifestyle interventions before progression to type 2 diabetes.

## Introduction

The prevalence of obesity has escalated dramatically in recent decades, throughout most of the Western world and much of the developing world [1] with a concomitant increase globally of the incidence of type 2 diabetes (T2D) [2]. Poor insulin sensitivity with increasing obesity typically leads to impaired glucose tolerance and pre-diabetes, which is estimated to affect 37% of the U.S. population [3]. Following the development of insulin resistance, the onset of T2D is prompted when the remaining functional beta-cells fail to compensate for the increased metabolic needs of the individual [4]. Genome wide association studies revealed that beta-cell mass and function is determined in part by genetics [5], suggesting that therapies for improving beta-cell function may be clinically relevant.

Proteomics has the potential to contribute significantly to the fields of nutrition and medical research [6, 7], with proteomic analysis of plasma and serum having potential to identify biomarkers of various conditions and diseases, including T2D [8–10]. Cathepsin D, leptin, interleukin 1 receptor antagonist, tissue plasminogen activator, renin, hepatocyte growth factor and fatty acid binding protein 4 have been identified as biomarkers of insulin resistance (HOMA-IR) in two non-diabetic community cohorts (n = 1367) [11]. Retinol binding protein (RBP4) was increased in fasting plasma samples of individuals with impaired glucose tolerance (n = 11) compared to healthy controls (n = 44) [12]. Plasminogen activator inhibitor-1 (PAI-1) was linked to T2D in a number of observational studies, with increased levels of PAI-1 in individuals with T2D [13, 14].

The identification of protein biomarkers for early detection of decreased beta-cell function could highlight the requirement for interventions to prevent or postpone T2D progression. The down-regulation of chaperone proteins and elongation factor 2 have been associated with high glucose-induced beta-cell dysfunction, and the downregulation of intracellular signalling, potentially decreasing insulin secretion [15]. Insulin resistance may develop following down-regulation of proteins involved in the control of insulin synthesis [16]. Studies in FoxO-deficient mice and their controls identified a proteomic signature following a mixed arginine-glucose tolerance test consisting of proteins involved in oxidoreductase activity, neuronal stress, and clotting, which correlated with the early onset of beta-cell dysfunction in rodents [17]. An unbiased iTRAQ (isobaric tag for relative and absolute quantification) was performed to assess the release of plasma proteins in response to a mixed arginine/glucose tolerance test, prior and post onset of hyperglycaemia, in FoxO-deficient mice and their controls. Firstly, an increase in plasma proteins regulating the oxidoreductive state was observed in healthy mice, while no increase was observed in the FoxO knockout mice. Secondly, both SNca and Park7, plasma proteins which represent neuronal stress, were increased in comparison to a decrease in both protein concentrations in FoxO knockouts. Lastly, proteins involved in clotting were elevated in plasma of FoxO knockout mice, for example Fga, Fgb, Fgg, Vwf, F13A1 and F13B [17]. Five of the identified proteins were increased before defects in insulin secretion were observed. In summary, the findings suggest that a reduction in the identified antioxidant proteins is an early marker of beta-cell dysfunction, however further investigation and confirmation in humans will be key to validating the proteins as potential biomarkers of beta-cell dysfunction.

In summary, proteomics in the study of pancreatic beta-cell function has potential to enhance early detection of reduced beta-cell function and monitor T2D onset. The current paper represents a subsequent step in the investigation of factors related to beta-cell function, using a proteomics dataset containing information on 1,129 protein levels [18]. The most routine method of assessing pancreatic beta-cell function is the oral glucose tolerance test (OGTT), however this method is time consuming and is burdensome for the participant. A single ideal biomarker may not exist for assessment of beta-cell function, therefore evaluating a panel of multiple biomarkers, preferably from a single fasting blood sample, may be required. The objectives of this study are to identify protein signatures and pathways related to pancreatic beta-cell function in fasting blood samples and confirm findings where possible.

## Materials and methods

### Study population and design

This research uses data and samples from the Metabolic Challenge Study (MECHE), which is part of a national research project by the Joint Irish Nutrigenomics Organisation (JINGO: www.ucd.ie/jingo/). The MECHE study recruited 214 healthy participants aged between 18–60 y and individuals were informed about the purpose of the study and the experimental procedures which would be involved, prior to giving written consent. Good health was defined as the absence of any known chronic or infectious disease and this was verified by a number of fasting blood tests. Details of the study have been published elsewhere [19–22]. Ethical approval was obtained from the Research Ethics Committees in University College Dublin (LS-08-43-Gibney-Ryan) and the study was performed according to the Declaration of Helsinki. The study was registered at clinicaltrials.gov under NCT01172951.

Baseline blood samples were collected on the morning of the study visits following an overnight fast. Participants underwent an OGTT or an oral lipid tolerance test according to the guidelines set by the World Health Organisation/International Diabetes Federation. Venous blood samples were taken before (0 min) and during the OGTT at set time-points (10, 20, 30, 60, 90 and 120 min), and serum and plasma samples were collected as previously described [19–22]. Details of the analytes and methods used are previously reported, along with the measurement of cytokines and hormones [19]. For the present study, participants with proteomic data obtained from a baseline serum sample and who completed an OGTT were included (n = 100).

### Assessment of pancreatic beta-cell function

Two specific measures of beta-cell function were assessed. Beta-cell function was calculated as the ratio of the incremental insulin to glucose response over the first 30 min of the OGTT (ΔInsulin_30_/ΔGlucose_30_). Beta-cell function was adjusted for HOMA-IR ((ΔInsulin_30_ /ΔGlucose_30_)/HOMA-IR). The oral disposition index (DI), which takes into account insulin sensitivity, was calculated for all participants ((ΔInsulin_30_/ΔGlucose_30_) x (1/Fasting Insulin)) [23].

### Measurement of proteomic data

The SOMAscan^™^ assay [24] was used to measure the levels of 1129 protein levels in fasting serum samples of MECHE participants. This technology has a dynamic range of more than eight logs, allowing quantitation of both low and high abundant proteins which might otherwise be missed. Proteins identified by SOMAmers are found in the blood as secreted (431), external membrane origin (275), and intracellular proteins (423). Proteins are often released from membranes by proteolytical cleavage and intracellular proteins may be released from cells as a part of normal or abnormal physiological cell turnover.

### Proteomic pathway and network analysis

Pathway over-representation analysis was performed with the human pathway collection from WikiPathways [25] (curated collection with 276 pathways downloaded on 26th January 2016) using PathVisio (version 3.2.4), a commonly used tool to create, visualise and analyse biological pathways [26]. Permuted p-value was calculated by performing a permutation test 1000 times followed by calculating a rank of the actual Z score compared to the permuted Z scores. The Z scores were calculated based on the changed proteins in a pathway out of the total proteins in the pathway that have been measured in the uploaded dataset. Proteins with p ≤ 0.05 were used for pathway analysis. The results of the pathway analysis were ranked based on the number of proteins significantly associated with beta-cell function measures. A pathway was considered important when the minimum number of differentially expressed proteins in the pathway was three, had a Z-score > 1.96, and had a permuted p < 0.05.

Networks were constructed between the proteins that were significantly associated with beta-cell function measures (p ≤ 0.01) and their direct interacting proteins. The networks were assembled by including protein-protein interaction (PPI) from the Human Protein Reference Database (HPRD). Data were extracted from the i2d database and self-loops and multiple interactions involving the same pairs of proteins were removed. The R package igraph was used to manipulate the PPI network. Visualisation was performed with Cytoscape 3.3. Biomart was used to convert UniProt ID to the HUGO Gene Nomenclature Committee (HGNC).

### Confirmation of beta-cell relationship in a separate cohort

Human calcineurin ELISAs were purchased from Elabscience (Patricell Limited, Nottingham United Kingdom). Baseline serum samples taken from 45 participants who underwent an OGTT as part of a Food for Health Ireland study were analysed for calcineurin levels.

### Cell culture and treatment

All chemicals were purchased from Sigma-Aldrich Ireland unless otherwise stated. Culture media and its related components were purchased from Gibco (Glasgow, UK). The BRIN-BD11 cell line was used in this study as it is a stable glucose responsive, insulin-secreting, beta-cell line [27]. The cells were maintained in RPMI-1640 containing 11.1 mM glucose, supplemented with 10% (v/v) foetal calf serum, 2 mM glutamine, 50 IU/mL penicillin, 0.05 mg/mL streptomycin and incubated at 37°C in a humidified atmosphere containing 5% C0_2_ and 95% air. For experimental treatments, cells were seeded at a density of 1.5 x 10^5^ cells per well in a 24-well plate for insulin secretion assays. Cells were allowed to attach for 24 h before treatment with 10, 20 or 50 ng/mL of rat IL-17F recombinant protein (Cambridge Biosciences) for 20 h. Acute insulin secretion assay was performed. Cell viability was assessed using WST-1 assay. Cells between passage 23–30 were used and all experiments were n = 4 unless otherwise stated.

### Acute insulin secretion

Following the 20 h treatment period, the culture medium was removed and the cells were washed with phosphate buffered saline (PBS). The cells were then incubated with Krebs-Ringer bicarbonate (KRB) buffer (115 mM NaCL, 1.28 mM CaCl_2_, 4.7 mM KCl, 1.2 mM KH_2_PO_4_, 1.2 mM MgSO_4_ 7H_2_O, 10 mM NaHC0_3_, 5 g/L BSA, all at pH 7.4) supplemented with 1.1 mM glucose for 40 min. The media was then replaced with KRB buffer containing 16.7 mM glucose plus 10 mM alanine, for 20 min. Following this 20 min incubation, the samples were transferred to Eppendorfs and centrifuged, before removing the supernatant and assaying for insulin content using a Mercodia Ultrasensitive Rat Insulin ELISA kit (Mercodia AB, Uppsala, Sweden).

### Statistical analysis

Analysis was performed using IBM SPSS Statistics 20. Data are expressed as means ± standard deviation. Data was examined using boxplots to identify extreme outliers for each protein, which was defined as Q_1_−3×IQR or above Q_3_ + 3×IQR. Pearsons correlation analysis assessed the association of proteins with beta-cell function measures, using p ≤ 0.01. Stepwise linear regression analysis determined the strongest relationships between beta-cell function measures and significant proteins (p ≤ 0.01 from correlation analysis). Statistical significance was evaluated using ANOVA with Bonferroni post-hoc tests for *in vitro* studies (p < 0.05). MetaboAnalyst 3.0 was used for ROC curve analysis. Participants were divided into tertiles based on their beta-cell function measures. ROC curves were produced to determine whether proteins of interest (p ≤ 0.01) could discriminate between the top 15 in the low and the top 15 participants in high tertile of beta-cell function. Pareto scaling was used and the random forest classification model was selected. The classification performance (sensitivity and specificity) of the samples was assessed by the area under the curve (AUC).

## Results

### Uncovering a proteomic signature related to beta-cell function

Participants (n = 100) who completed an OGTT with fasting proteomic profiles were included in this analysis. Baseline characteristics are summarised in Table 1. The cohort had an equal number of males and females and a mean BMI of 25.3 kg/m^2^. The study cohort were 95% Caucasian (3% Black, 1% Asian, 1% Mediterranean). Beta-cell function/HOMA-IR was significantly correlated with 22 proteins and the disposition index was significantly correlated with 17 proteins (p ≤ 0.01) (Table 2, S1 Fig). Protein concentrations are displayed across tertiles of beta-cell function measures in Table 3. Based on linear regression analysis, the strongest proteins associated with beta-cell function/HOMA-IR were IL-17F (β = -0.027) and beta-endorphin (β = 0.039). Calcineurin and CRTAM were the strongest proteins associated with the disposition index (β = 0.005 and 0.005 respectively) (S1 Table).

**Table 1:** Baseline characteristics of MECHE cohort (n = 100).

Variable	Mean ± S.D.
Sex (m/f)	50/50
Age (y)	32 ± 11
Weight (kg)	76.64 ± 17.15
BMI (kg/m^2^)	25.3 ± 5.5
Waist (cm)	85.8 ± 15.6
WHR	0.85 ± 0.1
BP SYS (mm/Hg)	122.9 ± 12.8
BP DIA (mm/Hg)	74.7 ± 10.8
Glucose (mmol/L)	5.21 ± 0.58
Total cholesterol (mmol/L)	4.49 ± 0.96
HDL cholesterol (mmol/L)	1.32 ± 0.36
TAG (mmol/L)	1.06 ± 0.61
Insulin (μIU/mL)	8.72 ± 6.93
HOMA-IR	1.99 ± 1.57

All values are means ± standard deviation. BMI, Body Mass Index; WHR, Waist to Hip Ratio; BP SYS, Systolic Blood Pressure; BP DIA, Diastolic Blood Pressure; HDL, High Density Lipoprotein cholesterol; TAG, triglycerides; HOMA-IR, Homeostatic Model Assessment of Insulin Resistance

**Table 2:** Pearson’s correlation of proteins with beta-cell function/HOMA-IR and the disposition index.

**Beta-cell function/HOMA-IR Proteins**	**Gene**	**Coefficient**	**P**
Beta-Endorphin	POMC	0.285	0.005
Cripto-1 growth factor	TDGF1	-0.267	0.008
IL-17F	IL-17F	-0.272	0.006
IL-22	IL-22	-0.259	0.01
Junctional adhesion molecule B	JAM2	-0.269	0.007
Galectin 2	LGALS2	0.306	0.002
A disintegrin and metalloproteinase with thrombospondin motifs 13	ADAMS13	0.259	0.009
WAP, Kazal, immunoglobulin, Kunitz and NTR domain-containing protein 2	WFIKKN2	0.258	0.01
Carbonic Anhydrase X	CA10	-0.277	0.007
Complement factor MASP-3	MASP1	-0.262	0.009
Group IIE secretory phospholipase A2	PLA2G2E	0.305	0.002
Ephrin type-B receptor 4	EphB4	-0.272	0.006
Legumain	LGMN	-0.287	0.004
Mediator of RNA polymerase II transcription subunit 1	MED1	0.265	0.008
Mesothelin	MSLN	0.328	0.001
Calcineurin	PPP3R1	0.272	0.006
D-dimer	FGA	0.354	0.0003
Peroxiredoxin 5	PRDX5	0.266	0.008
Cytotoxic and regulatory T-cell molecule	CRTAM	0.344	0.001
DAP kinase-related apoptosis-inducing protein kinase 2	STK17B	-0.282	0.005
Limbic system-associated membrane protein	LSAMP	0.268	0.007
Stromelysin-2	MMP10	0.263	0.009
**Disposition Index Proteins**	**Gene**	**Coefficient**	**P**
Beta-Endorphin	POMC	0.29	0.004
Insulin-like growth factor-binding protein 3	IGFBP3	0.259	0.01
Cripto-1 growth factor	TDGF1	-0.284	0.004
Junctional adhesion molecule B	JAM2	-0.264	0.009
Galectin 2	LGALS2	0.325	0.001
Cathepsin B	CTSB	0.256	0.01
Carbonic Anhydrase X	CA10	-0.263	0.01
Stromelysin-2	MMP10	0.28	0.005
Peroxiredoxin 5	PRDX5	0.273	0.007
Cytotoxic and regulatory T-cell molecule	CRTAM	0.367	0.001
Complement factor MASP-3	MASP1	-0.257	0.01
Ephrin type-B receptor 4	EphB4	-0.276	0.005
Legumain	LGMN	-0.277	0.005
DAP kinase-related apoptosis-inducing protein kinase 2	STK17B	-0.281	0.005
Calcineurin	PPP3R1	0.283	0.004
D-dimer	FGA	0.353	0.00035
Mesothelin	MSLN	0.348	0.001

Results obtained from Pearsons correlation, with correlation coefficients and p ≤ 0.01 presented.

**Table 3:** Protein concentrations across tertiles of beta-cell function measures.

**Beta-cell function/HOMA-IR related proteins**	**T1 (4.57 ± 2.07 pmol/mmol)**	**T2 (10.27 ± 1.70 pmol/mmol)**	**T3 (23.67 ± 8.16 pmol/mmol)**	**P**
Beta-Endorphin	488.8 ± 78.99	502.3 ± 49.68	554.4 ± 140.92	0.021
Cripto-1 growth factor	605.8 ± 58.07	593.3 ± 42.91	571.0 ± 40.91	0.015
IL-17F	888.8 ± 92.82	850.0 ± 64.94	834.8 ± 90.68	0.029
IL-22	470.7 ± 55.60	459.8 ± 51.09	439.9 ± 46.80	0.055
JAM-B	2857.4 ± 315.83	2765.3 ± 337.85	2606.3 ± 285.67	0.007
Galectin-2	950.8 ± 122.81	986.5 ± 106.39	1064.1 ± 154.43	0.002
ADAMS13	3488.5 ± 634.12	3724.8 ± 803.32	3976.6 ± 931.83	0.051
WFIKKN2	4470.5 ± 846.64	4898.6 ± 1036.92	5120.2 ± 1095.08	0.030
Carbonic Anhydrase X	672.6 ± 67.27	653.2 ± 49.11	648.2 ± 45.63	0.091
MASP-3	5498.5 ± 778.32	5190.6 ± 715.11	4995.6 ± 840.04	0.034
PLA2G2E	360.4 ± 30.03	375.9 ± 35.09	388.3 ± 47.64	0.015
EphB4	20856.3 ± 2139.79	20002.2 ± 2374.49	19132.2 ± 3287.01	0.034
LGMN	9492.1 ± 986.24	8905.2 ± 952.61	8626.7 ± 1292.85	0.006
MED-1	541.1 ± 38.16	555.8 ± 55.16	583.8 ± 76.79	0.014
Mesothelin	559.5 ± 83.80	599.2 ± 151.19	697.4 ± 361.64	0.055
Calcineurin	496.1 ± 95.12	556.3 ± 104.01	572.0 ± 133.11	0.017
D-dimer	2249.7 ± 386.28	2248.5 ± 391.11	2483.4 ± 530.12	0.053
Peroxiredoxin-5	573.9 ± 51.50	598.0 ± 66.48	629.8 ± 145.52	0.072
CRTAM	714.3 ± 75.25	747.0 ± 82.22	773.6 ± 167.40	0.142
STK17B	10299.7 ± 1222.92	9811.8 ± 1190.98	9412.9 ± 1541.83	0.028
LSAMP	1475.2 ± 230.80	1523.0 ± 182.98	1637.0 ± 279.46	0.018
Stromelysin-2	698.2 ± 134.68	664.6 ± 63.11	729.4 ± 94.73	0.038
**Disposition Index related proteins**	**T1 (1.08 ± 0.45 pmol/mmol)**	**T2 (2.30 ± 0.39 pmol/mmol)**	**T3 (5.33 ± 1.80 pmol/mmol)**	**P**
Beta-Endorphin	488.0 ± 78.17	499.0 ± 51.31	558.7 ± 138.90	0.009
IGFBP-3	1055.6 ± 234.27	1133.9 ± 159.66	1192.6 ± 212.11	0.028
Cripto-1 growth factor	605.9 ± 56.36	593.7 ± 43.10	570.5 ± 42.83	0.013
JAM-B	2826.3 ± 313.07	2783.8 ± 350.27	2617.9 ± 287.18	0.025
Galectin 2	950.1 ± 124.59	991.8 ± 100.17	1059.2 ± 159.46	0.004
Cathepsin B	2145.2 ± 232.24	2148.3 ± 274.98	2219.3 ± 346.06	0.499
Carbonic Anhydrase X	673.2 ± 67.91	651.6 ± 50.66	649.3 ± 42.05	0.171
Stromelysin-2	703.4 ± 133.67	655.2 ± 58.50	734.1 ± 93.25	0.007
Peroxiredoxin-5	571.7 ± 50.69	600.5 ± 66.86	629.3 ± 145.30	0.062
CRTAM	718.9 ± 73.34	742.0 ± 81.96	775.1 ± 168.90	0.167
MASP-3	5468.0 ± 768.34	5183.3 ± 760.26	5033.6 ± 827.38	0.079
EphB4	20733.6 ± 2014.20	20023.6 ± 2565.95	19232.8 ± 3217.17	0.078
LGMN	9454.4 ± 995.39	8932.0 ± 1039.30	8636.8 ± 1234.69	0.011
STK17B	10264.8 ± 1203.18	9800.0 ± 1264.52	9459.9 ± 1519.56	0.053
Calcineurin	490.4 ± 91.73	547.6 ± 99.45	586.7 ± 133.39	0.002
D-dimer	2234.7 ± 385.76	2255.4 ± 397.06	2491.9 ± 521.38	0.037
Mesothelin	558.7 ± 82.67	607.8 ± 152.97	690.4 ± 363.48	0.076

Protein concentrations (relative fluorescence units) displayed as mean ± standard deviations across tertiles (T) of beta-cell functions measures. P-values obtained from one way ANOVA, overall P-value displayed.

### Discrimination between low and high beta-cell function using the identified protein signature

**Fig 1:**
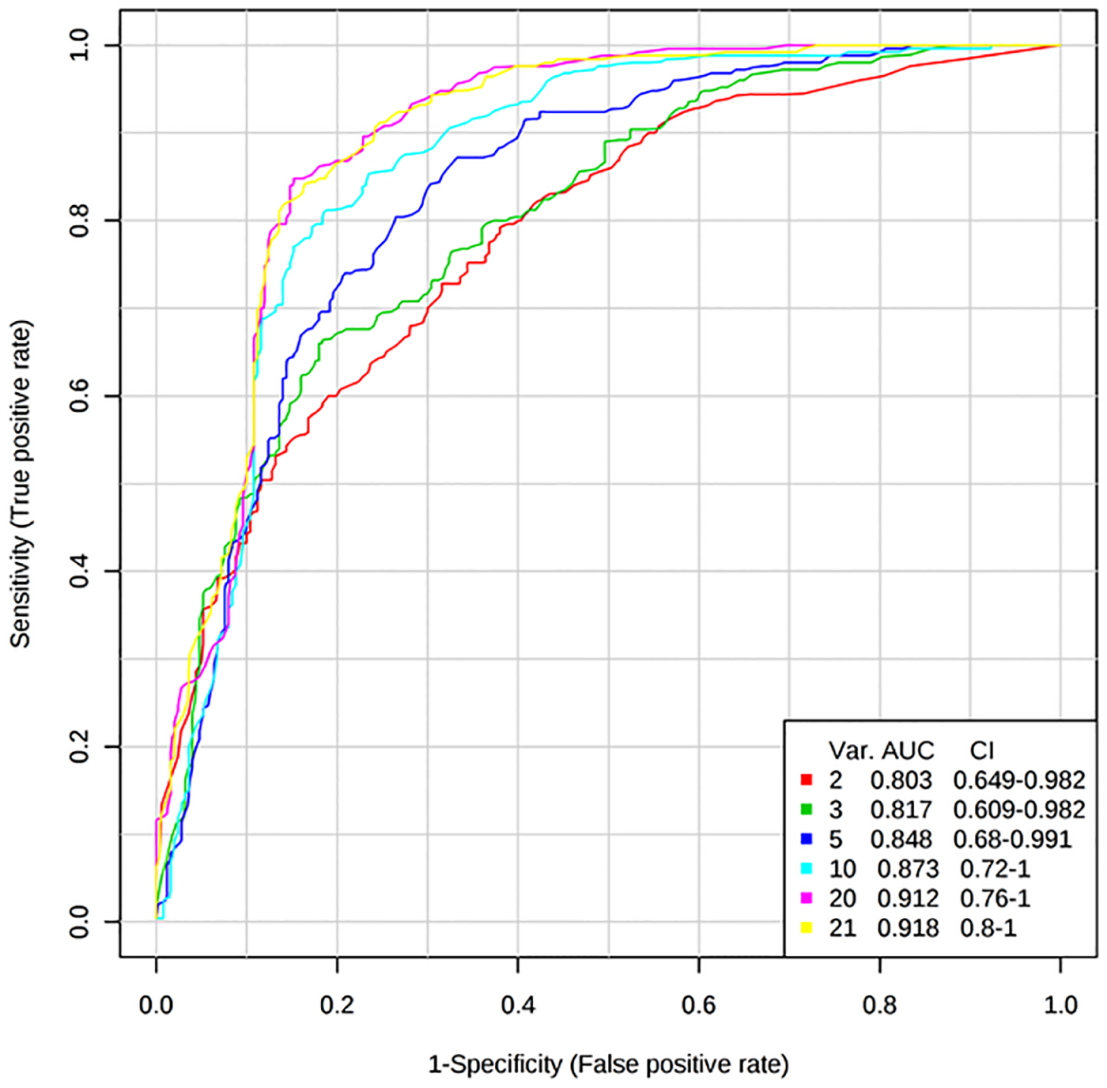
ROC curves for assessment of protein panel to discriminate between low and high disposition index measures. ROC curves to determine the predictive ability of the protein panel (17 proteins associated with the disposition index (p ≤ 0.01)), age, BMI, waist to hip ratio and fasting glucose, for classification of low and high disposition index values of MECHE participants (n = 30). Par scaling was used as a scaling method and random forest method was employed for classification of variables. Using all 21 variables, the best ROC curve was produced with an AUC of 0.918. AUC: area under the curve. Var: variable. CI: Confidence interval. Tertile 1: low beta-cell function Tertile 3: high beta-cell function.

**Fig 2:**
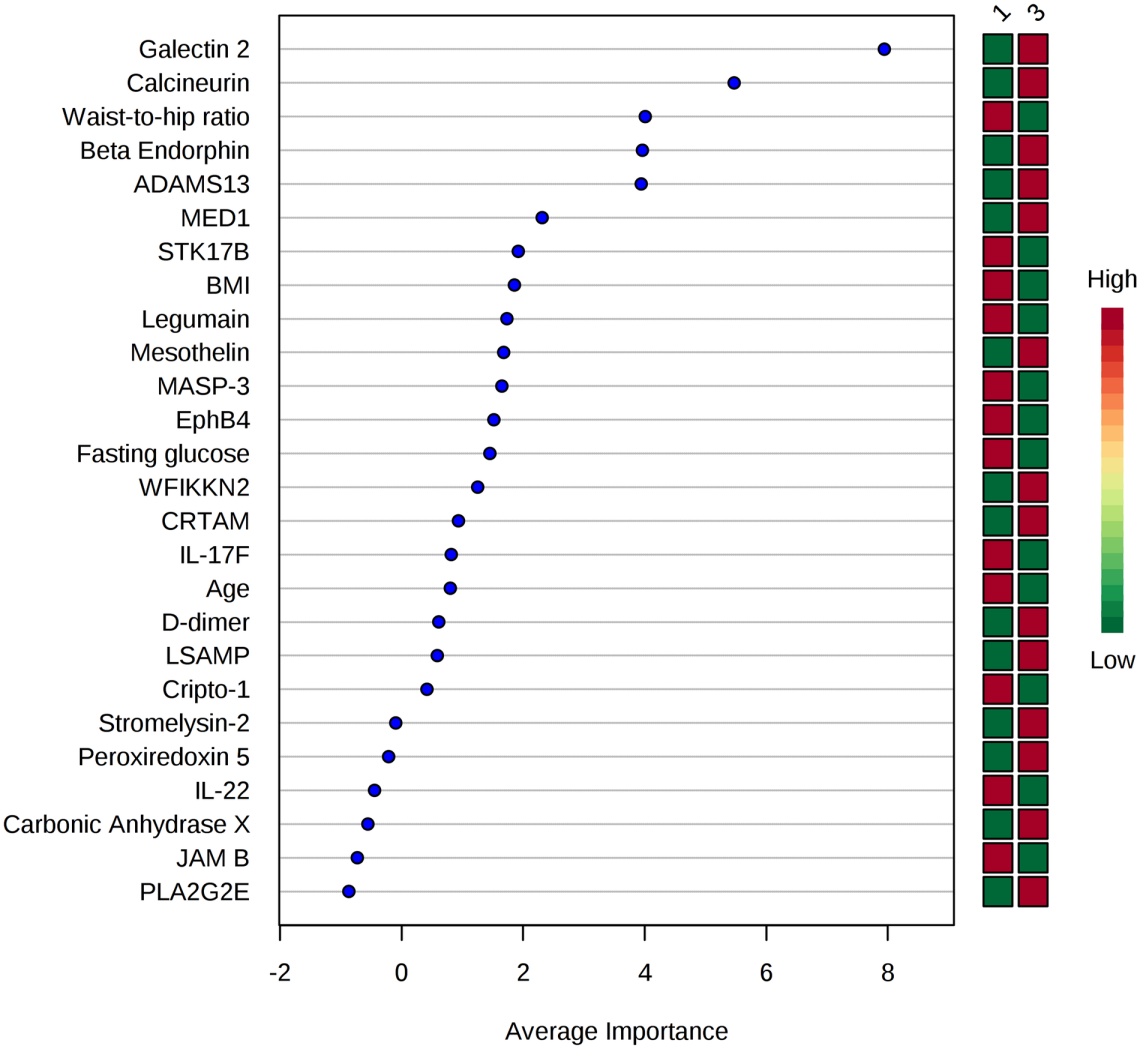
Features ranked by mean importance measure for the ROC curve analysis for beta-cell function/HOMA-IR. Determination of the predictive ability of the protein panel (22 proteins associated with beta-cell function/HOMA-IR (p ≤ 0.01)), age, BMI, waist to hip ratio and fasting glucose, for discrimination between low and high beta-cell function/HOMA-IR values of MECHE participants (n = 30). Par scaling was used and random forest classification method was selected. Using all 26 variables, the best ROC curve was produced with an AUC of 0.913. AUC: area under the curve. **1**: Tertile 1- low beta-cell function **3:** Tertile 3- high beta-cell function. Green filled square: Low concentration/value Red filled square: High concentration/value Green filled square/ Red filled square: Positive association with beta-cell function/HOMA-IR Red filled square/Green filled Square: Inverse association with beta-cell function/HOMA-IR.

ROC curve analysis used a multivariate model to examine the predictive accuracy of proteins to discriminate between low and high beta-cell function. Biochemical and demographic data were also included, to compare the clinical variables ability with the identified proteins ability to discriminate between high and low beta-cell function. For the disposition index, a ROC curve was produced using the panel of 17 proteins (Table 2), age, BMI, waist-to-hip ratio and fasting glucose, which discriminated between low and high disposition index values, resulting in an AUC of 0.918 (Fig 1, S2 Fig). Similarly, a ROC curve was produced using the panel of 22 proteins (Table 2), age, BMI, waist to hip ratio and fasting glucose, and discriminated between low and high beta-cell function/HOMA-IR (AUC = 0.913) (S3 Fig). The contribution of individual variables by mean importance measure to ROC curve analysis for beta-cell function/HOMA-IR is displayed in Fig 2.

Furthermore, using the identified sex, age and body fat mass associated proteins from previous analysis by our research group, potential confounding factors for the identified protein panels were examined [28]. For beta-cell function/HOMA-IR associated proteins, the protein WFIKKN2 was significantly inversely associated with body fat mass and LSAMP was significantly higher in females and inversely associated with fat mass (2 out of 22 proteins also related to phenotypic parameters). For proteins associated with the disposition index, only one out of 17 proteins (IGFBP3) was also significantly associated with aging (1 out of 17 proteins impacted by a phenotypic parameter). Therefore no overall impact from phenotypic parameters on the identified proteomic panels was observed.

### Pathways and networks emerge as related to beta-cell function measures

Pathway analysis performed in PathVisio using the WikiPathways human collection of curated pathways revealed altered pathways for both beta-cell function measures (Table 4, S2 and S3 Tables, S4 Fig). A total of 16 pathways contained three or more proteins significantly associated with beta-cell function/HOMA-IR and 12 pathways with the disposition index (S2 and S3 Tables). Proteins with p ≤ 0.05 for Pearson correlation analysis with beta-cell function measures were used for pathway analysis (S4 and S5 Tables). Five of the 16 pathways associated with beta-cell function/HOMA-IR and two pathways related to the disposition index met the significance criteria (p < 0.05), had a Z score >1.96, and had three or more significantly associated proteins (Table 4). The percentage of total gene products displayed in Table 4, S2 and S3 Tables refers to percentage coverage of each pathway by SOMAscan assay.

**Table 4:** Pathways significantly related to beta-cell function measures.

WikiPathway ID	Pathway	Positive	Measured by SOMAscan assay	Z Score	P	% of total gene products measured in pathway	Significant proteins
**Beta-cell function/ HOMA-IR**							
WP558	Complement and Coagulation Cascades	8	40	2.57	0.02	64.4	TFPI, a1-antitrypsin, kininogen HMW, C7, MASP3, C1s, coagulation factor IX/ coagulation factor IXab, C3a
WP545	Complement Activation	5	17	3.03	0.03	77.3	C7, C3a, C1s, Masp3, Properdin
WP2064	Neural Crest Differentiation	5	18	2.88	0.01	17.8	Cadherin 2, Cadherin 6, FGFR2, MIA, HDAC8
WP2858	Ectoderm Differentiation	4	17	2.17	0.03	11.3	Cadherin 6, FGFR2, MCP1, GIB
WP3414	Initiation of transcription and translation elongation at the HIV-1 LTR	3	3	5.59	<0.001	6.3	HDAC8, Calcineurin, Calcineurin Ba
**Disposition Index**							
WP558	Complement and Coagulation Cascades	8	40	2.98	0.01	64.4	TFPI, a1-antitrypsin, kininogen HMW, C7, MASP3, C1s, coagulation factor IX/ coagulation factor IXab, C3a
WP545	Complement Activation	4	17	2.47	0.03	77.3	C7, C3a, C1s, MASP3

Pathways obtained from pathway statistics using PathVisio software, using the curated WikiPathways directory. Sorted by number of differentially expressed proteins (positive) in pathway (measured by SOMAscan assay). P-value is permuted, p < 0.05 is considered significant. Percentage of total gene products refers to % coverage of pathway by SOMAscan assay.

**Fig 3:**
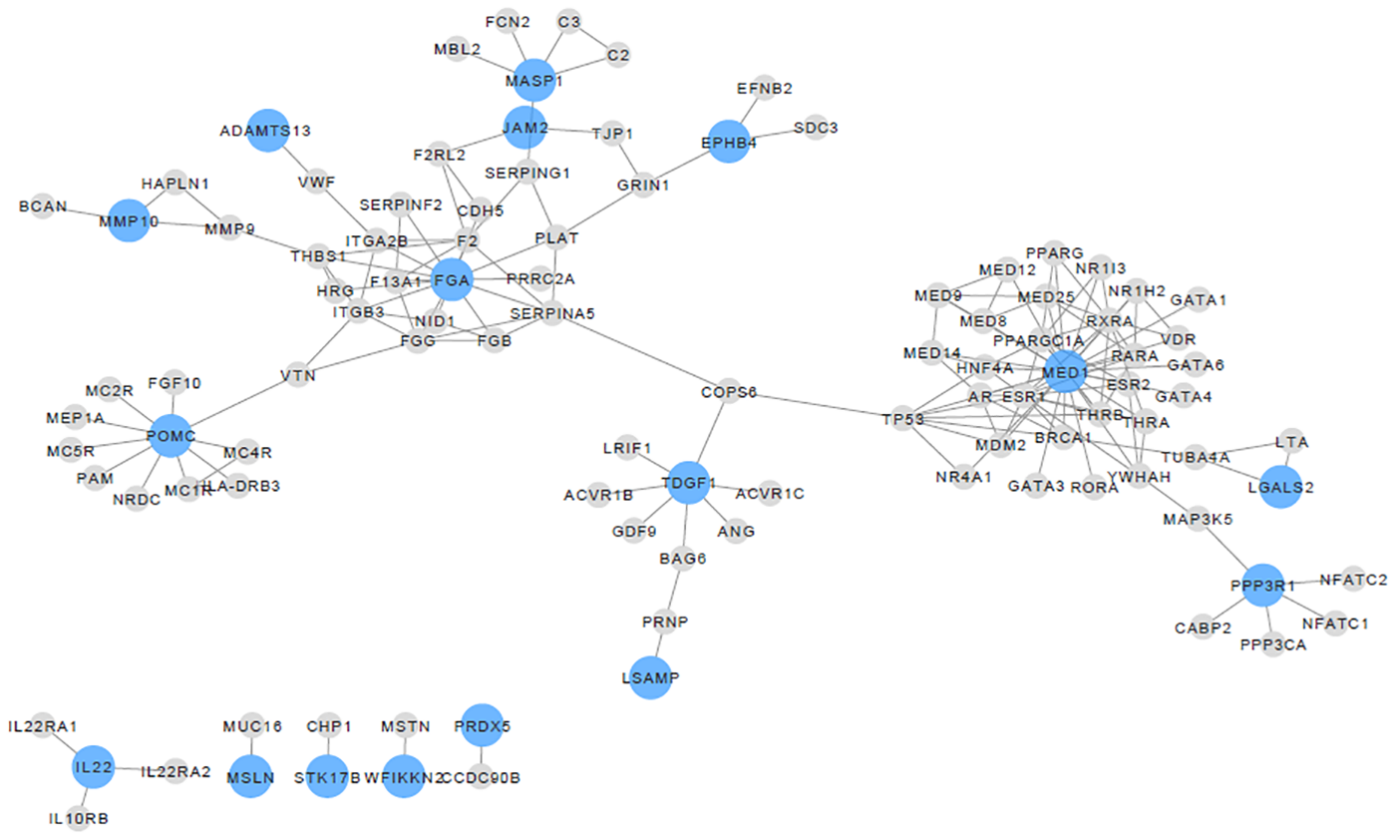
Network displaying proteins significantly associated with beta-cell function/HOMA-IR and their direct interactions. The nodes represent proteins and the edges represent protein-protein interactions. Proteins significantly related to beta-cell function/HOMA-IR are in blue (p ≤ 0.01) and grey nodes are their direct interactions. 17/22 proteins significantly associated with beta-cell function/HOMA-IR have direct interactions.

**Fig 4:**
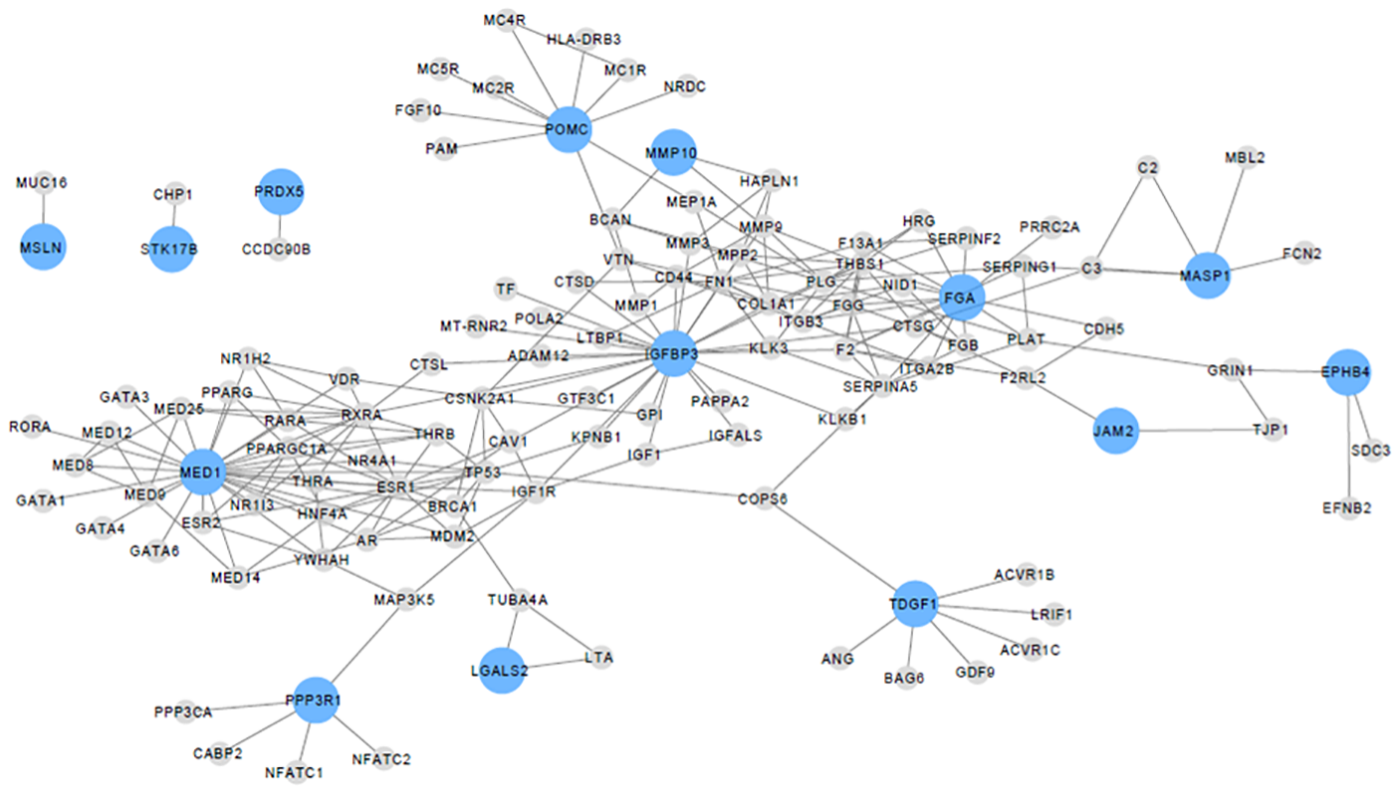
Network displaying proteins significantly associated with the disposition index and their direct interactions. The nodes represent proteins and the edges represent protein-protein interactions. Proteins significantly related with the disposition index are in blue (p ≤ 0.01) and grey nodes are their direct interactions. 13/17 proteins significantly associated with the disposition index have direct interactions.

Networks were constructed between beta-cell function proteins and their direct interacting proteins. Two networks were constructed for both beta-cell function/HOMA-IR and the disposition index. Fig 3 displays the network between beta-cell function/HOMA-IR proteins and direct interacting proteins with p ≤ 0.01 while Fig 4 displays the network between disposition index proteins and direct interacting proteins with p ≤ 0.01. Seventeen out of 22 significant proteins (Table 2) for beta-cell function/HOMA-IR had direct interactions, while 13 out of 17 proteins (Table 3) for disposition index had direct interactions.

### Confirmation of beta-cell functions relationship with calcineurin and IL-17F

**Fig 5:**
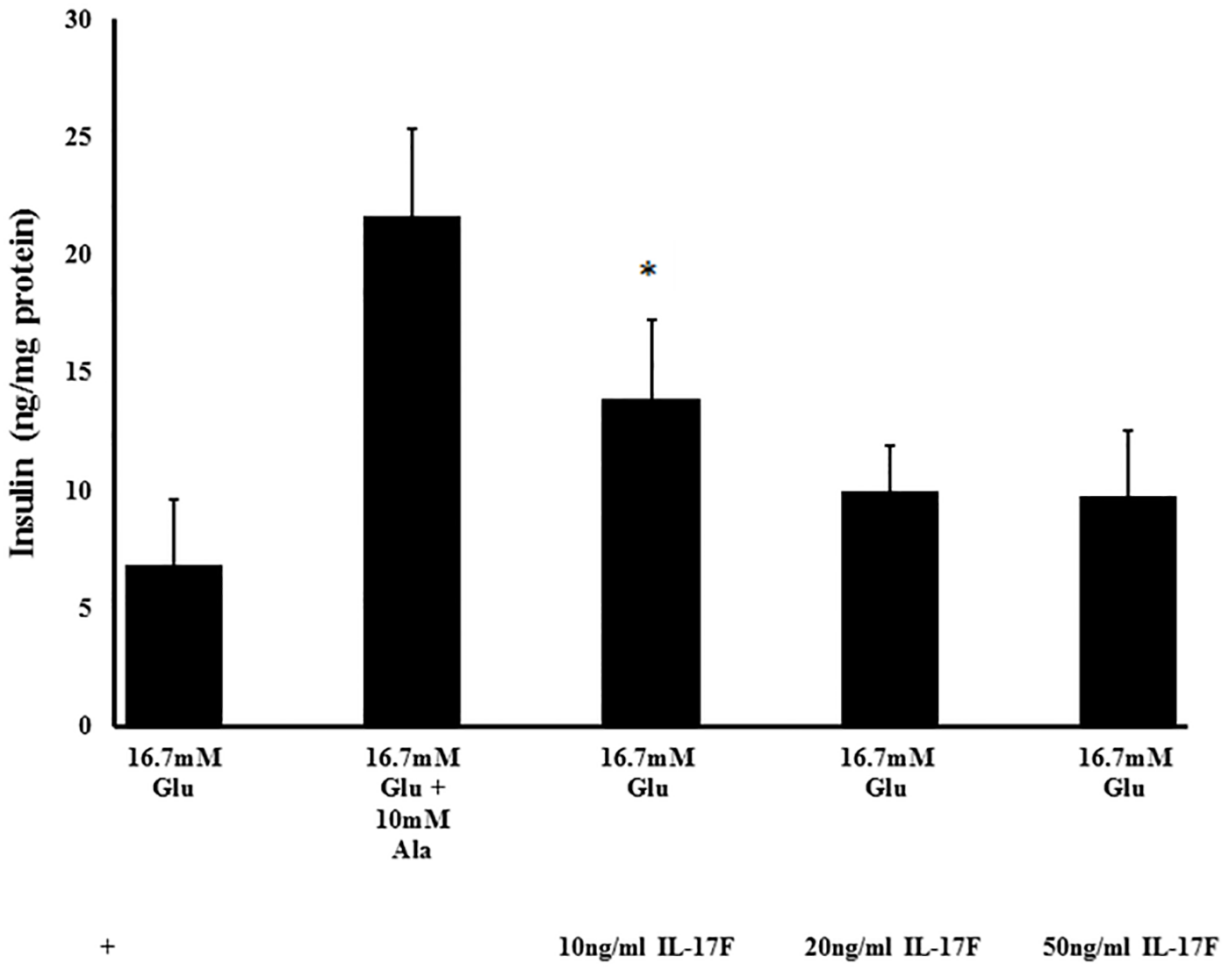
The effect of 20 h treatment with IL-17F on insulin secretion in BRIN-BD11 cell line. Values are mean ± standard deviation (n = 4). *p < 0.05. ANOVA was applied across groups with post-hoc Bonferroni test for comparison of treatments (10, 20 and 50 ng/mL IL-17F) with 16.7mM glucose (control). Cells were incubated for 20 h with 10, 20 and 50 ng/mL IL-17F, and stimulated with 16.7 mM glucose to determine insulin secretion. Overall p = 0.000041. *p = 0.043 where treatment with 10 ng/mL IL-17F is significantly increased in comparison to control of 16.7mM glucose.

The Food for Health (FHI) cohort (n = 45) had a mean age of 53 y and a mean BMI of 31.6 kg/m^2^ (S6 Table). The relationship between calcineurin and beta-cell function was successfully confirmed in this independent cohort, with correlation coefficients of 0.299 and 0.316 for beta-cell function/HOMA-IR and disposition index respectively (p = 0.046 and p = 0.035 respectively). To assess the effects of exposure to IL-17F on pancreatic beta-cells, BRIN-BD11 cells were incubated with IL-17F for 20 h. No loss of cell viability was observed during the incubation period (S5 Fig). Following exposure to IL-17F, cells treated with the lower concentration (10 ng/mL) displayed a significant increase in glucose stimulated insulin secretion in comparison to the control (p = 0.043). No significant effect on insulin secretion was observed with the higher concentrations of IL-17F (Fig 5).

## Discussion

The present study identified a proteomic signature related to pancreatic beta-cell function, capable of discriminating between low and high beta-cell function measures. Early detection of decreased pancreatic beta-cell function would allow for the implementation of nutritional and lifestyle interventions to reduce the risk of T2D [29]. Importantly, the ROC curve analysis highlighted the potential of the identified panel for use in a clinical setting. In addition, pathway analysis was performed to explore the biological basis of the altered proteomic signatures, with the complement and coagulation cascades pathway emerging as the top pathway associated with beta-cell function.

The SOMAscan assay analyses serum protein levels across a wide dynamic range and has been used to identify proteins associated with metabolic processes involved in T2D [30–33]. Belongie and colleagues identified proteomic and miRNA biomarkers of beta-cell function in fasting samples from 43 participants who had impaired glucose tolerance (IGT) and 43 controls who maintained normal glucose tolerance over a three year period [33]. Beta-cell function was assessed by calculating the beta-cell glucose sensitivity index and the oral glucose insulin sensitivity index, both of which were derived from data from OGTTs. The top proteins differentially expressed in participants with decreased beta-cell function following the three year follow up were adiponectin, alpha1-antitrypsin and endocan. In our study, both adiponectin and alpha1-antitrypsin were identified as meeting the p < 0.05 criteria. Belongie and colleagues also included clinical variables in combination with proteins to predict beta-cell function, and their models identified glucose during OGTT (time-points 60, 90, 120 min) as the top predictors, followed by the proteins of interest. In our study, proteomic signatures in conjunction with demographic and biochemical variables displayed high predictive ability for discriminating between low and high beta-cell function measures. Galectin 2 and calcineurin emerged as the top variables to discriminate between both high and low measures of beta-cell function. The proteins displayed greater discriminatory ability than clinical variables, such as fasting glucose levels and BMI. Galectin 2 (LGALS2) is a beta-galactoside binding protein and was recently discovered as the first endogenous CD14/TLR4 ligand which induces a pro-inflammatory, non-arteriogenic phenotype in monocytes/macrophages [34]. The waist-to-hip ratio which was identified as strongly related to beta-cell function in previous analysis [18], also emerged as influential in classifying individuals between low and high beta-cell function. Overall, the ROC curve analysis promotes the potential use of the panel in a clinical setting and should be further developed and validated.

Based on regression results, calcineurin, beta-endorphin, and CRTAM were positively associated with beta-cell function measures based on regression results whereas IL-17F was negatively associated. Calcineurin emerged as a highly influential feature in ROC curve analysis in the MECHE cohort, a result replicated in an independent human cohort. Calcineurin was previously reported to have a relationship with beta-cell function [35]. Calcineurin/nuclear factor of activated T-cells (NFAT) signalling regulates several factors that control regulation of beta-cell function, demonstrating a potential avenue for enhanced understanding of the pathogenesis of T2D [36]. Protein kinase R-like endoplasmic reticulum kinase was observed to regulate calcineurin with the proteins working in combination to modulate insulin secretion and calcium signalling in pancreatic beta-cells [37]. The results presented in this analysis strengthen the positive relationship of calcineurin with beta-cell function.

Beta-endorphin was an important variable in ROC curve analysis for discrimination between high and low beta-cell function, in addition to having a strong association with beta-cell function in regression analysis. Beta-endorphin was first linked with beta-cell function and T2D risk in the 1980s [38, 39]. Beta-endorphin (2.5 mg IV bolus) administered to non-insulin dependent individuals with T2D resulted in increased plasma levels of insulin at 90 min with a decrease in plasma glucose [40]. In contrast, relatively little is known about CRTAM with respect to pancreatic beta-cell function and T2D risk. CRTAM is a transmembrane protein that is located on the cell surface of activated invariant natural killer T (iNKT) cells, CD8+, and CD4+ T cells [41]. CRTAM expression was linked with pro-inflammatory profiles and cellular activities, such as adhesion, cytotoxicity, and Th1 and Th17 cytokine production [42]. A recent study reported lower iNKT cell counts in type 1 diabetics and CRTAM was identified as a marker of activated iNKT lymphocytes [43]. Despite the potential role of CRTAM in the inflammatory immune response, its role in relation to T2D is relatively unknown. The current analysis observed a positive association of CRTAM with beta-cell function, a novel finding and warrants further investigation.

An inverse relationship was observed between IL-17F and beta-cell function. Recently, Th17 signature cytokines IL-21, IL-22 and IL-17F were identified as important indicators of T2D, with IL-17F explaining some of the variance in HbA1c [44]. This group also analysed production of TNFα in a number of different peripheral blood mononuclear cells (PBMC) in the presence or absence of IL-17A and/or IL-17F blocking antibodies. IL-17F neutralisation reduced production of TNFα by both CD41 and CD81 T cells [44], suggesting that a Th17 signature associated with T2D regulates downstream TNFα production. The *in vitro* analysis presented here showed that IL-17F also modulated insulin secretion, with a low dose of IL-17F eliciting an increase in insulin secretion with no observed effects at the higher doses. This result is consistent with the initial negative association of IL-17F and beta-cell function measures.

Pathway analysis was used to analyse the main biological processes involved with beta-cell function based on levels of serum proteins. Complement and coagulation cascades pathway was the top pathway in which eight proteins were significantly associated with beta-cell function. The complement system is a major component of both the innate and adaptive immune responses and the coagulation pathway has been previously associated with T2D by others [45]. While the complement and coagulation cascade pathway is implicated in various disease states [46] the proteins altered in the pathway may be specific to the biological process or disease under investigation. In the current analysis, C3a, C7 and alpha1-antitrypsin were positively associated with beta-cell function whereas TFPI, MASP 1, Kininogen HMW, C1s and coagulation factor IX were negatively associated with beta-cell function. Previous literature reported increased expression of C1s in primary adipose cells from abdominal subcutaneous adipose tissue of individuals with insulin resistance [47]. The IL-17 signalling pathway contained three proteins associated with beta-cell function/HOMA-IR and the disposition index, with IL-17A and IL-17F negatively and IL-17sR positively associated with beta-cell function measures. IL-17A and IL-17F are the most studied proteins of the IL-17 cytokine family, with both proteins pro-inflammatory under most of the conditions studied and may participate in driving autoimmunity [48].

The involvement of proteins with metabolic alterations or disease can be successfully explored using protein interaction networks, due to protein-protein interactions representing connections between cell components and processes [49]. Any alterations in protein-protein interactions may lead to disturbances in cellular processes. Due to interactions of cellular components and co-dependencies, the pathogenesis of disease states are rarely due to disruptions in one protein, often alterations are present in an intracellular network [50]. Network analysis of the plasma proteome data in this study identified a network related to beta-cell function. FGA (D-dimer) and MED1 (Mediator of RNA polymerase II transcription subunit 1) are key regulators in the beta-cell function/HOMA-IR network, while IGFBP3 appears to be a key protein in the disposition index network. Beta-endorphin and galectin 2 are also in these networks, reinforcing their strong relationship with beta-cell function measures. The network analysis complemented the ROC curve analysis in highlighting the benefit of using a protein panel instead of a single protein for assessment of beta-cell function.

Strengths of the present study include direct assessment of fasting proteomic data with specific beta-cell function measures obtained during an OGTT, confirmation of calcineurin in an independent cohort, and also effects of added IL-17F in cell assays. *In vitro* results mirrored the inverse relationship of IL-17F with beta-cell function and provided an opportunity to examine the direct effects on insulin secretion. Confirmation of the relationship between calcineurin with beta-cell function in the FHI human cohort, who were slightly older and had a higher BMI strengthens the association of calcineurin and beta-cell function measures. Limitations of the present study include the study population being limited to Irish participants. Expansion of this research to non-Irish and non-European cohorts would be beneficial in order to fully translate the research findings to the global population. In addition, the SOMAscan assay does not allow absolute quantification of protein levels and data were expressed in relative fluorescence units. Absolute quantification may be necessary to set reference values and translate results to the clinic.

## Conclusions

The SOMAscan assay was used to assess levels of 1129 proteins in 100 individuals which identified a proteomic signature related to pancreatic beta-cell function. The ROC curve analysis highlighted the potential of the panel for use in a clinical setting for early detection of beta-cell dysfunction. Early detection of decreased pancreatic beta-cell function would allow for implementation of dietary and lifestyle interventions before progression into T2D status. Further work is needed to replicate this signature in a larger cohort of individuals with varying degrees of beta-cell function.

## Supporting information

S1 Table Linear regression analysis to determine a proteomic signature related to beta-cell function measures. Results obtained from stepwise linear regression analysis, with unstandardised beta coefficients and 95% confidence intervals. IL-17F; interleukin 17F, CRTAM; cytotoxic and regulatory t-cell adhesion molecule.
(DOCX)

S2 Table List of pathways related to beta-cell function/HOMA-IR. Pathways obtained from pathway statistics using PathVisio software, using the curated WikiPathways directory. Sorted by number of differentially expressed proteins in pathway. P-value is permuted. Percentage of total gene products refers to % coverage of pathway by SOMAscan assay.
(DOCX)

S3 Table List of pathways related to the disposition index. Pathways obtained from pathway statistics using PathVisio software, using the curated WikiPathways directory. Sorted by number of differentially expressed proteins in pathway. P-value is permuted. Percentage of total gene products refers to % coverage of pathway by SOMAscan assay.
(DOCX)

S4 Table Pearson’s correlation of proteins with beta-cell function/HOMA-IR. Results obtained from Pearson’s correlation analysis, with correlation coefficients and p ≤ 0.05 presented.
(DOCX)

S5 Table Pearson’s correlation of proteins with the disposition index. Results obtained from Pearson’s correlation analysis, with correlation coefficients and p ≤ 0.05 presented.
(DOCX)

S6 Table Baseline characteristics FHI cohort (n = 45). All values are means ± standard deviation. BMI, Body Mass Index; BP SYS, Systolic Blood Pressure; BP DIA, Diastolic Blood Pressure; HOMA-IR, Homeostatic Model Assessment of Insulin Resistance; BCF/HOMA IR, beta-cell function adjusted by HOMA IR.
(DOCX)

S1 Fig Scatterplots of proteins related to Disposition Index from Pearson’s correlation analysis (p ≤ 0.01). Protein concentrations displayed in relative fluorescence units (RFU’s).
(DOCX)

S2 Fig The receiver operating characteristic curve analysis to assess the predictive ability of ability of the protein panel (17 proteins associated with the disposition index (p ≤ 0.01)), age, BMI, waist to hip ratio and fasting glucose, for classification of low and high disposition index values of MECHE participants (n = 30). Par scaling was used and random forest classification method was selected. Using all 21 variables, the best ROC curve was produced with an AUC of 0.918. AUC: area under the curve. **A)** Features ranked by mean importance measure for the receiver operating characteristic curve. Tertile 1: low beta-cell function Tertile 3: high beta-cell function Green filled square: Low concentration/value Red filled square: High concentration/value Green filled square/Red filled square: Positive association with beta-cell function/HOMA-IR Red filled square/Green filled square: Inverse association with beta-cell function/HOMA-IR **B)** Probabilities of predicted belonging to the low beta-cell function. Overall 26 participants were classified correctly into low beta-cell function (1) and high beta-cell function (3). Four participants were not correctly classified. **C)** An overview of the predictive accuracies using different amounts of variables. Using all 21 variables, the model is 83% accurate in predicting participants to have low or high beta-cell function.
(DOCX)

S3 Fig The receiver operating characteristic curve to assess the predictive ability of the protein panel (22 proteins associated with the beta-cell function/HOMA-IR (p ≤ 0.01)), age, BMI, waist to hip ratio and fasting glucose, for classification of low and high beta-cell function/ HOMA-IR values of MECHE participants (n = 30). Par scaling was used and random forest classification method was selected. Using all 26 variables, the best ROC curve was produced with an AUC of 0.913. AUC: area under the curve.
(DOCX)

S4 Fig IL-17 signalling pathway obtained from WikiPathways displaying proteins significantly associated with beta-cell function measures. Green filled square: Up-regulated with increasing beta-cell functionRed filled square: Down-regulated with increasing beta-cell functionYellow Filled square: Measured but no association
(DOCX)

S5 Fig Cell viability of BRIN-BD11 cells following treatment with different concentrations of IL-17F for 20 h (n = 4). Values are expressed as mean ± SD. No significant differences were observed. Sodium azide is the negative control.
(DOCX)

S1 Data SOMA data.xlsx. The SOMA data used in the study. Each row represents an individual.
(XLSX)

## References

[1] Ng M, Fleming T, Robinson M, Thomson B, Graetz N, Margono C, et al Global, regional, and national prevalence of overweight and obesity in children and adults during 1980–2013: a systematic analysis for the Global Burden of Disease Study 2013. Lancet. 2014;384(9945):766–81. Epub 2014/06/02. 10.1016/S0140-6736(14)60460-8 . 24880830 PMC4624264

[2] Whiting DR, Guariguata L, Weil C, Shaw J. IDF diabetes atlas: global estimates of the prevalence of diabetes for 2011 and 2030. Diabetes Res Clin Pract. 2011;94(3):311–21. Epub 2011/11/15. 10.1016/j.diabres.2011.10.029 . 22079683

[3] Bansal N. Prediabetes diagnosis and treatment: A review. World J Diabetes. 2015;6(2):296–303. Epub 2015/03/20. 10.4239/wjd.v6.i2.296 . 25789110 PMC4360422

[4] Cerf ME. Beta cell dysfunction and insulin resistance. Front Endocrinology. 2013;4:37 10.3389/fendo.2013.00037 23542897 PMC3608918

[5] Doria A, Patti ME, Kahn CR. The emerging genetic architecture of type 2 diabetes. Cell Metab. 2008;8(3):186–200. Epub 2008/09/03. 10.1016/j.cmet.2008.08.006 . 18762020 PMC4267677

[6] Oller Moreno S, Cominetti O, Nunez Galindo A, Irincheeva I, Corthesy J, Astrup A, et al The differential plasma proteome of obese and overweight individuals undergoing a nutritional weight loss and maintenance intervention. Proteomics Clinical applications. 2017 Epub 04/04. 10.1002/prca.201600150 . 28371297

[7] Runau F, Arshad A, Isherwood J, Norris L, Howells L, Metcalfe M, et al Potential for proteomic approaches in determining efficacy biomarkers following administration of fish oils rich in omega-3 fatty acids: application in pancreatic cancers. Nutr Clin Pract. 2015;30(3):363–70. Epub 2015/01/27. 10.1177/0884533614567337 . 25616520

[8] Riaz S. Study of Protein Biomarkers of Diabetes Mellitus Type 2 and Therapy with Vitamin B1. J Diabetes Res. 2015;2015:150176 Epub 2015/08/15. 10.1155/2015/150176 . 26273663 PMC4530253

[9] Scott EM, Carter AM, Findlay JB. The application of proteomics to diabetes. Diab Vasc Dis Res. 2005;2(2):54–60. Epub 2005/11/25. 10.3132/dvdr.2005.009 . 16305059

[10] Mukherjee P, Mani S. Methodologies to decipher the cell secretome. Biochim Biophys Acta. 2013;1834(11):2226–32. Epub 2013/02/05. 10.1016/j.bbapap.2013.01.022 . 23376189 PMC3652893

[11] Nowak C, Sundström J, Gustafsson S, Giedraitis V, Lind L, Ingelsson E, et al Protein Biomarkers for Insulin Resistance and Type 2 Diabetes Risk in Two Large Community Cohorts. Diabetes. 2015;65(1):276 10.2337/db15-0881 26420861 PMC5860375

[12] Umeno A, Yoshino K, Hashimoto Y, Shichiri M, Kataoka M, Yoshida Y. Multi-Biomarkers for Early Detection of Type 2 Diabetes, Including 10- and 12-(Z,E)-Hydroxyoctadecadienoic Acids, Insulin, Leptin, and Adiponectin. PloS one. 2015;10(7):e0130971 10.1371/journal.pone.0130971 26132231 PMC4488492

[13] Al-Hamodi Z, Ismail IS, Saif-Ali R, Ahmed KA, Muniandy S. Association of plasminogen activator inhibitor-1 and tissue plasminogen activator with type 2 diabetes and metabolic syndrome in Malaysian subjects. Cardiovascular diabetology. 2011;10:23 Epub 2011/03/19. 10.1186/1475-2840-10-23 . 21414238 PMC3064636

[14] Yarmolinsky J, Bordin Barbieri N, Weinmann T, Ziegelmann PK, Duncan BB, Ines Schmidt M. Plasminogen activator inhibitor-1 and type 2 diabetes: a systematic review and meta-analysis of observational studies. Scientific reports. 2016;6:17714 Epub 01/28. 10.1038/srep17714 . 26813008 PMC4728395

[15] Maris M, Ferreira GB, D'Hertog W, Cnop M, Waelkens E, Overbergh L, et al High glucose induces dysfunction in insulin secretory cells by different pathways: a proteomic approach. J Proteome Res. 2010;9(12):6274–87. Epub 2010/10/15. 10.1021/pr100557w . 20942503

[16] El Ouaamari A, Zhou J, Liew CW, Shirakawa J, Dirice E, Gedeon N, et al Compensatory Islet Response to Insulin Resistance Revealed by Quantitative Proteomics. Journal of proteome research. 2015;14(8):3111–22. Epub 2015/07/08. 10.1021/acs.jproteome.5b00587 . 26151086 PMC4615688

[17] Kuo T, Kim-Muller JY, McGraw TE, Accili D. Altered Plasma Profile of Antioxidant Proteins as an Early Correlate of Pancreatic beta Cell Dysfunction. J Biol Chem. 2016; 291(18):9648–56. Epub 2016/02/27. 10.1074/jbc.M115.702183 . 26917725 PMC4850302

[18] Curran AM, Ryan MF, Drummond E, Gibney ER, Gibney MJ, Roche HM, et al Uncovering Factors Related to Pancreatic Beta-Cell Function. PloS one. 2016;11(8):e0161350 10.1371/journal.pone.0161350 27536890 PMC4990237

[19] Morris C, O’Grada C, Ryan M, Roche HM, Gibney MJ, Gibney ER, et al Identification of differential responses to an oral glucose tolerance test in healthy adults. PloS one. 2013;8(8):e72890 Epub 2013/08/31. 10.1371/journal.pone.0072890 . 23991163 PMC3749984

[20] O’Gorman A, Morris C, Ryan M, O’Grada CM, Roche HM, Gibney ER, et al Habitual dietary intake impacts on the lipidomic profile. J Chromatogr B Analyt Technol Biomed Life Sci. 2014;966:140–6. Epub 2014/02/26. 10.1016/j.jchromb.2014.01.032 . 24565891

[21] Ryan MF, O’Grada CM, Morris C, Segurado R, Walsh MC, Gibney ER, et al Within-person variation in the postprandial lipemic response of healthy adults. Am J Clin Nutr. 2013;97(2):261–7. Epub 2013/01/04. 10.3945/ajcn.112.047936 . 23283501

[22] Wallace M, Morris C, O’Grada CM, Ryan M, Dillon ET, Coleman E, et al Relationship between the lipidome, inflammatory markers and insulin resistance. Mol Biosyst. 2014;10(6):1586–95. Epub 2014/04/10. 10.1039/c3mb70529c . 24714806

[23] Utzschneider KM, Prigeon RL, Faulenbach MV, Tong J, Carr DB, Boyko EJ, et al Oral disposition index predicts the development of future diabetes above and beyond fasting and 2-h glucose levels. Diabetes Care. 2009;32(2):335–41. Epub 2008/10/30. 10.2337/dc08-1478 . 18957530 PMC2628704

[24] Gold L, Ayers D, Bertino J, Bock C, Bock A, Brody EN, et al Aptamer-based multiplexed proteomic technology for biomarker discovery. PloS one. 2010;5(12):e15004 Epub 2010/12/18. 10.1371/journal.pone.0015004 . 21165148 PMC3000457

[25] Kelder T, van Iersel MP, Hanspers K, Kutmon M, Conklin BR, Evelo CT, et al WikiPathways: building research communities on biological pathways. Nucleic acids research. 2012;40(Database issue):1301–7. Epub 2011/11/19. 10.1093/nar/gkr1074 . 22096230 PMC3245032

[26] Kutmon M, van Iersel MP, Bohler A, Kelder T, Nunes N, Pico AR, et al PathVisio 3: an extendable pathway analysis toolbox. PLoS Comput Biol. 2015;11(2):e1004085 Epub 2015/02/24. 10.1371/journal.pcbi.1004085 . 25706687 PMC4338111

[27] McClenaghan NH, Barnett CR, Ah-Sing E, Abdel-Wahab YH, O’Harte FP, Yoon TW, et al Characterization of a novel glucose-responsive insulin-secreting cell line, BRIN-BD11, produced by electrofusion. Diabetes. 1996;45(8):1132–40. Epub 1996/08/01. . 8690162 10.2337/diab.45.8.1132

[28] Curran AM, Fogarty Draper C, Scott-Boyer M-P, Valsesia A, Roche HM, Ryan MF, et al Sexual Dimorphism, Age, and Fat Mass Are Key Phenotypic Drivers of Proteomic Signatures. Journal of proteome research. 2017;16(11):4122–33. 10.1021/acs.jproteome.7b00501 28950061

[29] The Diabetes Prevention Program (DPP) Research Group. The Diabetes Prevention Program (DPP). Diabetes care. 2002;25(12):2165 12453955 10.2337/diacare.25.12.2165 PMC1282458

[30] Hathout Y. Proteomic methods for biomarker discovery and validation. Are we there yet? Expert Rev Proteomics. 2015;12(4):329–31. Epub 2015/07/18. 10.1586/14789450.2015.1064771 . 26186709

[31] Kiddle SJ, Steves CJ, Mehta M, Simmons A, Xu X, Newhouse S, et al Plasma protein biomarkers of Alzheimer’s disease endophenotypes in asymptomatic older twins: early cognitive decline and regional brain volumes. Transl Psychiatry. 2015;5:e584 Epub 2015/06/17. 10.1038/tp.2015.78 mc4490288. 26080319 PMC4490288

[32] Menni C, Kiddle SJ, Mangino M, Vinuela A, Psatha M, Steves C, et al Circulating Proteomic Signatures of Chronological Age. J Gerontol A Biol Sci Med Sci. 2015;70(7):809–16. Epub 2014/08/16. 10.1093/gerona/glu121 . 25123647 PMC4469006

[33] Belongie KJ, Ferrannini E, Johnson K, Andrade-Gordon P, Hansen MK, Petrie JR. Identification of novel biomarkers to monitor β-cell function and enable early detection of type 2 diabetes risk. PloS one. 2017;12(8):e0182932 10.1371/journal.pone.0182932 28846711 PMC5573304

[34] Yıldırım C, Vogel DYS, Hollander MR, Baggen JM, Fontijn RD, Nieuwenhuis S, et al Galectin-2 Induces a Proinflammatory, Anti-Arteriogenic Phenotype in Monocytes and Macrophages. PloS one. 2015;10(4):e0124347 10.1371/journal.pone.0124347 25884209 PMC4401781

[35] Renstrom E, Ding WG, Bokvist K, Rorsman P. Neurotransmitter-induced inhibition of exocytosis in insulin-secreting beta cells by activation of calcineurin. Neuron. 1996;17(3):513–22. Epub 1996/09/01. . 8816714 10.1016/s0896-6273(00)80183-x

[36] Heit JJ, Apelqvist AA, Gu X, Winslow MM, Neilson JR, Crabtree GR, et al Calcineurin/NFAT signalling regulates pancreatic beta-cell growth and function. Nature. 2006;443(7109):345–9. Epub 2006/09/22. 10.1038/nature05097 . 16988714

[37] Wang R, McGrath BC, Kopp RF, Roe MW, Tang X, Chen G, et al Insulin secretion and Ca2+ dynamics in beta-cells are regulated by PERK (EIF2AK3) in concert with calcineurin. J Biol Chem. 2013;288(47):33824–36. Epub 2013/10/12. 10.1074/jbc.M113.503664 . 24114838 PMC3837125

[38] Feldman M, Kiser RS, Unger RH, Li CH. Beta-endorphin and the endocrine pancreas. Studies in healthy and diabetic human beings. N Engl J Med. 1983;308(7):349–53. Epub 1983/02/17. 10.1056/NEJM198302173080701 . 6296674

[39] Reid RL, Yen SS. beta-Endorphin stimulates the secretion of insulin and glucagon in humans. J Clin Endocrinol Metab. 1981;52(3):592–4. Epub 1981/03/01. 10.1210/jcem-52-3-592 . 7007411

[40] Reid RL, Sandler JA, Yen SS. Beta-endorphin stimulates the secretion of insulin and glucagon in diabetes mellitus. Metabolism. 1984;33(3):197–9. Epub 1984/03/01. . 6319955 10.1016/0026-0495(84)90035-0

[41] Yeh JH, Sidhu SS, Chan AC. Regulation of a late phase of T cell polarity and effector functions by Crtam. Cell. 2008;132(5):846–59. Epub 2008/03/11. 10.1016/j.cell.2008.01.013 . 18329370

[42] Kennedy J, Vicari AP, Saylor V, Zurawski SM, Copeland NG, Gilbert DJ, et al A molecular analysis of NKT cells: identification of a class-I restricted T cell-associated molecule (CRTAM). J Leukoc Biol. 2000;67(5):725–34. Epub 2000/05/16. . 10811014 10.1002/jlb.67.5.725

[43] Beristain-Covarrubias N, Canche-Pool E, Gomez-Diaz R, Sanchez-Torres LE, Ortiz-Navarrete V. Reduced iNKT cells numbers in type 1 diabetes patients and their first-degree relatives. Immunity, inflammation and disease. 2015;3(4):411–9. Epub 2016/01/07. 10.1002/iid3.79 . 26734463 PMC4693717

[44] Ip B, Cilfone NA, Belkina AC, DeFuria J, Jagannathan-Bogdan M, Zhu M, et al Th17 cytokines differentiate obesity from obesity-associated type 2 diabetes and promote TNFalpha production. Obesity (Silver Spring). 2016;24(1):102–12. Epub 2015/11/19. 10.1002/oby.21243 . 26576827 PMC4688084

[45] Calimlioglu B, Karagoz K, Sevimoglu T, Kilic E, Gov E, Arga KY. Tissue-Specific Molecular Biomarker Signatures of Type 2 Diabetes: An Integrative Analysis of Transcriptomics and Protein-Protein Interaction Data. Omics: a journal of integrative biology. 2015;19(9):563–73. Epub 2015/09/09. 10.1089/omi.2015.0088 . 26348713

[46] Mäkinen V-P, Civelek M, Meng Q, Zhang B, Zhu J, Levian C, et al Integrative Genomics Reveals Novel Molecular Pathways and Gene Networks for Coronary Artery Disease. PLoS genetics. 2014;10(7):e1004502 10.1371/journal.pgen.1004502 25033284 PMC4102418

[47] Zhang J, Wright W, Bernlohr DA, Cushman SW, Chen X. Alterations of the classic pathway of complement in adipose tissue of obesity and insulin resistance. Am J Physiol Endocrinol Metab. 2007;292(5):E1433–40. Epub 2007/01/25. 10.1152/ajpendo.00664.2006 . 17244723

[48] Gaffen SL. Structure and signalling in the IL-17 receptor family. Nat Rev Immunol. 2009;9(8):556–67. Epub 2009/07/04. 10.1038/nri2586 . 19575028 PMC2821718

[49] Rao VS, Srinivas K, Sujini GN, Kumar GNS. Protein-Protein Interaction Detection: Methods and Analysis. International Journal of Proteomics. 2014;2014:147648 10.1155/2014/147648 24693427 PMC3947875

[50] Barabási A-L, Gulbahce N, Loscalzo J. Network Medicine: A Network-based Approach to Human Disease. Nat Rev Genet. 2011;12(1):56–68. 10.1038/nrg2918 21164525 PMC3140052

