# Supplementary material for: A proteomic signature that reflects pancreatic beta-cell function
Source: PLoS One. 2018 Aug 30;13(8):e0202727. doi: 10.1371/journal.pone.0202727 (PMC6117012; doi:10.1371/journal.pone.0202727)
Supplement: S1 Table — Results obtained from stepwise linear regression analysis, with unstandardised beta coefficients and 95% confidence intervals. IL-17F; interleukin 17F, CRTAM; cytotoxic and regulatory t-cell adhesion molecule. (DOCX) [file pone.0202727.s001.docx]

**Online Supplementary Material**

**S1 Table. Linear regression analysis to determine a proteomic signature related to beta-cell function measures.**

| **Beta-cell function/HOMA-IR** | | | | **Disposition Index** | | | |
| --- | --- | --- | --- | --- | --- | --- | --- |
| **Variable** | **B** | ***P*** | **95% CI** | **Variable** | **B** | ***P*** | **95% CI** |
| Beta-Endorphin | 0.039 | 0.005 | (0.012, 0.065) | CRTAM | 0.0054 | 0.012 | (0.0013, 0.0096) |
| IL-17F | -0.027 | 0.013 | (-0.047, -0.006) | Calcineurin | 0.0047 | 0.012 | (0.0011, 0.0084) |

Results obtained from stepwise linear regression analysis, with unstandardised beta coefficients and 95% confidence intervals. IL-17F; interleukin 17F, CRTAM; cytotoxic and regulatory t-cell adhesion molecule
