# Supplementary material for: A proteomic signature that reflects pancreatic beta-cell function
Source: PLoS One. 2018 Aug 30;13(8):e0202727. doi: 10.1371/journal.pone.0202727 (PMC6117012; doi:10.1371/journal.pone.0202727)
Supplement: S2 Table — Pathways obtained from pathway statistics using PathVisio software, using the curated WikiPathways directory. Sorted by number of differentially expressed proteins in pathway. P-value is permuted. Percentage of total gene products refers to % coverage of pathway by SOMAscan assay. (DOCX) [file pone.0202727.s002.docx]

**S2 Table. List of pathways related to beta-cell function/HOMA-IR**

| Pathway and ID | Positive | | Measured by SOMAscan assay | Z | *P* | % of total gene products measured in pathway | | Significant proteins | |
| --- | --- | --- | --- | --- | --- | --- | --- | --- | --- |
| Complement and Coagulation Cascades (WP558) | 8 | | 40 | 2.57 | 0.02 | 64.4 | TFPI, a1 antitrypsin, kininogen hmw, c7, masp3,c1s, coagulation factor IX/ coagulation factor Ixab, c3a | |  |
| Complement Activation (WP545) | 5 | | 17 | 3.03 | 0.03 | 77.3 | C7, C3a, C1s, Masp3, Properdin | |  |
| Allograft Rejection (WP2328) | 5 | | 39 | 0.91 | 0.46 | 46.2 | IL22, IL17a, IL5, C3a, C7 | |  |
| Neural Crest Differentiation (WP2064) | 5 | | 18 | 2.88 | 0.01 | 17.8 | Cadherin 2, Cadherin 6, FGFR2, MIA, HDAC8 | |  |
| Regulation of Actin Cytoskeleton (WP51) | 4 | | 44 | 0.08 | 0.94 | 29.5 | Moesin, FGF20, FGF9, FGFR2 | |  |
| Ectoderm Differentiation (WP2858) | 4 | | 17 | 2.17 | 0.03 | 11.3 | Cadherin 6, FGFR2, MCP1, GIB | |  |
| Blood Clotting Cascade (WP272) | 3 | | 15 | 1.55 | 0.10 | 68.2 | Coagulation factor IX/ coagulation factor Ixab, D-dimer, Fibrinogen g-chain dimer | |  |
| Matrix Metalloproteinases (WP129) | 3 | | 17 | 1.31 | 0.14 | 54.8 | MMP-8, MMP-13, MMP-10 | |  |
| Oncostatin M Signalling Pathway (WP2374) | 3 | | 30 | 0.24 | 0.92 | 47.7 | MCP1, MMP13, TYK2 | |  |
| IL-17 signalling pathway (WP2112) | | 3 | 14 | 1.69 | 0.08 | 41.9 | L17A, IL17F, IL17sR | |  |
| Spinal Cord Injury (WP2431) | 3 | | 46 | -0.55 | 0.56 | 37.9 | MCP-1, calcineurin, groa-a | |  |
| Integrated Pancreatic Cancer Pathway (WP2377) | 3 | | 70 | -1.38 | 0.19 | 36.3 | HDAC8, Neurotrophin 3, IGFBP3, | |  |
| Selenium Micronutrient Network (WP15) | 3 | | 27 | 0.44 | 0.67 | 33.7 | Peroxiredoxin 5, MCP1, CRP | |  |
| MAPK signalling Pathway (WP382) | 3 | | 52 | -0.79 | 0.44 | 27.4 | Calcineurin B a, Calcineurin, MK12 | |  |
| BDNF signalling pathway (WP2380) | 3 | | 39 | -0.24 | 0.82 | 27.1 | Neurotrophin 3, Cadherin 2, CAMK1 | |  |
| Initiation of transcription and translation elongation at the HIV-1 LTR (WP3414) | 3 | | 3 | 5.59 | <0.001 | 6.3 | HDAC8, Calcineurin, Calcineurin Ba | |  |

Pathways obtained from pathway statistics using PathVisio software, using the curated WikiPathways directory. Sorted by number of differentially expressed proteins in pathway. P-value is permuted. Percentage of total gene products refers to % coverage of pathway by SOMAscan assay.
