# Supplementary material for: A proteomic signature that reflects pancreatic beta-cell function
Source: PLoS One. 2018 Aug 30;13(8):e0202727. doi: 10.1371/journal.pone.0202727 (PMC6117012; doi:10.1371/journal.pone.0202727)
Supplement: S3 Table — Pathways obtained from pathway statistics using PathVisio software, using the curated WikiPathways directory. Sorted by number of differentially expressed proteins in pathway. P-value is permuted. Percentage of total gene products refers to % coverage of pathway by SOMAscan assay. (DOCX) [file pone.0202727.s003.docx]

**S3 Table. List of pathways related to the disposition index**

| **Pathway** | **Positive** | **Measured** **by SOMAscan assay** | **Z** | ***P*** | **% of total gene products measured in pathway** | **Significant proteins** |
| --- | --- | --- | --- | --- | --- | --- |
| Complement and Coagulation Cascades (WP558) | 8 | 40 | 2.98 | 0.01 | 64.4 | TFPI, a1-antitrypsin, kininogen HMW, C7, MASP3, C1s, coagulation factor IX/ coagulation factor IXab, C3a |
| Allograft Rejection (WP2328) | 5 | 39 | 1.22 | 0.14 | 46.2 | IL22, IL17a, IL5, C3a, C7 |
| Complement Activation **(**WP545**)** | 4 | 17 | 2.47 | 0.03 | 77.3 | C7, C3a, C1s, MASP3 |
| Spinal Cord Injury (WP2431) | 4 | 46 | 0.26 | 0.77 | 37.9 | MCP-1, calcineurin, galectin-3, TLR4 |
| BDNF signalling pathway (WP2380) | 4 | 39 | 0.61 | 0.51 | 27.1 | Neurotrophin 3, TrkB, Cadherin 2, CAMK1 |
| Blood Clotting Cascade (WP272) | 3 | 15 | 1.8 | 0.08 | 68.2 | coagulation factor IX/ coagulation factor IXab, D-dimer, Fibrinogen g-chain dimer |
| Matrix Metalloproteinases **(**WP129**)** | 3 | 17 | 1.55 | 0.10 | 54.8 | MMP-8, MMP-13, MMP-10 |
| Oncostatin M Signalling Pathway **(**WP2374**)** | 3 | 30 | 0.48 | 0.86 | 47.7 | MCP1, MMP13, TYK2 |
| IL-17  signalling pathway (WP2112) | 3 | 14 | 1.94 | 0.07 | 41.9 | IL17A, IL17F, IL17sR |
| Regulation of toll-like receptor signalling pathway (WP1449) | 3 | 40 | -0.05 | 0.98 | 25.2 | TLR4, TLR4s, MK12, |
| Neural Crest Differentiation **(**WP2064**)** | 3 | 18 | 1.44 | 0.12 | 17.8 | Cadherin 2, Cadherin 6, FGFR-2 |
| Ectoderm Differentiation (WP2858) | 3 | 17 | 1.55 | 0.07 | 11.3 | Cadherin 6, FGFR2, MCP1 |

Pathways obtained from pathway statistics using PathVisio software, using the curated WikiPathways directory. Sorted by number of differentially expressed proteins in pathway. P-value is permuted. Percentage of total gene products refers to % coverage of pathway by SOMAscan assay.
