# Supplementary material for: A proteomic signature that reflects pancreatic beta-cell function
Source: PLoS One. 2018 Aug 30;13(8):e0202727. doi: 10.1371/journal.pone.0202727 (PMC6117012; doi:10.1371/journal.pone.0202727)
Supplement: S4 Table — Results obtained from Pearson’s correlation analysis, with correlation coefficients and p ≤ 0.05 presented. (DOCX) [file pone.0202727.s004.docx]

**S4 Table. Pearson’s correlation of proteins with beta-cell function/HOMA-IR**

| Target name | Mean ± SD | Coefficient | *P* |
| --- | --- | --- | --- |
| SHBG | 5049.1 ± 3460.03 | 0.247 | 0.015 |
| CRTAM | 745.3 ± 117.41 | 0.344 | 0.001 |
| DOPA decarboxylase | 338.9 ± 40.27 | 0.211 | 0.036 |
| LGMN | 9007.0 ± 1134.63 | -0.287 | 0.004 |
| C7 | 1252.2 ± 247.82 | 0.231 | 0.021 |
| STK17B | 9841.1 ± 1362.40 | -0.282 | 0.005 |
| Calcineurin | 541.6 ± 115.46 | 0.272 | 0.006 |
| EphB4 | 19997.0 ± 2710.56 | -0.272 | 0.006 |
| IL-17F | 857.8 ± 85.82 | -0.272 | 0.006 |
| D-dimer | 2323.2 ± 447.41 | 0.354 | <0.001 |
| Carbonic Anhydrase X | 658.3 ± 55.49 | -0.277 | 0.007 |
| JAM-B | 27435 ± 327.79 | -0.269 | 0.007 |
| Cripto | 590.3 ± 49.56 | -0.267 | 0.008 |
| Mesothelin | 616.0 ± 229.88 | 0.328 | 0.001 |
| Peroxiredoxin-5 | 600.5 ± 98.17 | 0.266 | 0.008 |
| MK12 | 452.1 ± 57.74 | 0.246 | 0.016 |
| Glutamate carboxypeptidase | 1186.9 ± 108.38 | -0.223 | 0.026 |
| WFIKKN2 | 4830.4 ± 1024.93 | 0.258 | 0.009 |
| IL-22 | 457.0 ± 52.39 | -0.259 | 0.010 |
| CD70 | 2595.4 ± 240.24 | -0.241 | 0.016 |
| Siglec-3 | 1789.4 ± 717.91 | 0.251 | 0.011 |
| Cadherin-6 | 2832.3 ± 593.75 | -0.206 | 0.039 |
| C1s | 6397.8 ± 900.34 | -0.252 | 0.012 |
| PLA2G2E | 374.7 ± 39.51 | 0.305 | 0.002 |
| Apo D | 17490.3 ± 2646.13 | -0.215 | 0.032 |
| BAFF Receptor | 2156.9 ± 625.19 | -0.247 | 0.013 |
| Kallikrein 14 | 20524.8 ± 2905.26 | -0.198 | 0.048 |
| BCMA | 3529.1 ± 801.67 | 0.245 | 0.014 |
| Moesin | 1263.3 ± 294.35 | 0.200 | 0.050 |
| FABPE | 420.6 ± 28.61 | -0.247 | 0.015 |
| NRX3B | 884.2 ± 145.47 | 0.213 | 0.034 |
| Cathepsin H | 3412.3 ± 271.72 | -0.241 | 0.016 |
| OCAD1 | 1451.4 ± 214.56 | -0.231 | 0.021 |
| CONA1 | 304.7 ± 34.71 | 0.245 | 0.016 |
| SAP | 31264.1 ± 6456.03 | -0.219 | 0.029 |
| H6ST1 | 318.2 ± 19.55 | -0.243 | 0.016 |
| MCP-1 | 566.5 ± 105.15 | 0.222 | 0.028 |
| OBCAM | 7978.3 ± 1108.16 | -0.240 | 0.016 |
| Cadherin-2 | 2390.2 ± 348.98 | 0.234 | 0.026 |
| RANK | 775.8 ± 125.49 | -0.242 | 0.016 |
| TYK2 | 8608.7 ± 1074.95 | -0.239 | 0.017 |
| CD109 | 5443.5 ± 1161.20 | 0.199 | 0.047 |
| IL-17 A | 326.2 ± 21.73 | -0.242 | 0.018 |
| Gro-a | 1283.3 ± 144.58 | -0.216 | 0.034 |
| IL-17 sR | 520.5 ± 166.20 | 0.233 | 0.019 |
| GIB | 1069.4 ± 223.53 | 0.205 | 0.042 |
| Beta-Endorphin | 514.6 ± 99.64 | 0.285 | 0.005 |
| Cathepsin B | 2170.7 ± 287.27 | 0.233 | 0.019 |
| PTP-1B | 1850.3 ± 450.52 | -0.233 | 0.020 |
| ADAMS13 | 3729.9 ± 815.76 | 0.259 | 0.009 |
| DYRK3 | 5506.2 ± 860.29 | -0.230 | 0.021 |
| GFRa-1 | 1410.2 ± 279.24 | -0.218 | 0.029 |
| Coagulation Factor IX | 10085.9 ± 1479.34 | -0.230 | 0.022 |
| Epithelial cell kinase | 2423.0 ± 399.78 | 0.228 | 0.022 |
| CRP | 27817.2 ± 19073.41 | -0.200 | 0.046 |
| IGFBP-1 | 1821.8 ± 1149.64 | 0.227 | 0.024 |
| UFM1 | 2547.4 ± 861.73 | 0.206 | 0.046 |
| PTHrP | 714.5 ± 103.49 | 0.231 | 0.024 |
| MED-1 | 560.0 ± 60.64 | 0.265 | 0.008 |
| CD226 | 1538.9 ± 155.51 | 0.228 | 0.026 |
| C3a | 747.4 ± 375.92 | 0.199 | 0.048 |
| FGF-20 | 507.2 ± 70.91 | 0.223 | 0.027 |
| WKFN1 | 582.1 ± 148.81 | -0.220 | 0.027 |
| CBG | 1122.1 ± 100.74 | -0.226 | 0.028 |
| DBNL | 1150.2 ± 180.48 | 0.248 | 0.016 |
| Nr-CAM | 14455.9 ± 3695.54 | 0.220 | 0.029 |
| PDE11 | 2650.2 ± 251.23 | -0.251 | 0.012 |
| PDGF-CC | 673.2 ± 455.40 | 0.225 | 0.030 |
| MMP-10 | 697.4 ± 103.49 | 0.263 | 0.009 |
| LSAMP | 1544.9 ± 241.32 | 0.268 | 0.007 |
| Glypican 3 | 963.9 ± 239.87 | 0.218 | 0.031 |
| Properdin | 36607.3 ± 6450.96 | -0.206 | 0.040 |
| Neurotrophin-3 | 326.1 ± 37.92 | 0.222 | 0.031 |
| Adiponectin | 2073.5 ± 861.24 | 0.250 | 0.012 |
| KI2L4 | 983.3 ± 214.30 | 0.207 | 0.042 |
| Coagulation Factor IXab | 10085.9 ± 1479.34 | -0.214 | 0.033 |
| FGF9 | 476.5 ± 46.48 | 0.201 | 0.050 |
| a1-Antitrypsin | 547.3 ± 142.83 | 0.236 | 0.018 |
| MMP-13 | 1082.5 ± 191.20 | 0.215 | 0.034 |
| TFPI | 36034.1 ± 9761.14 | -0.244 | 0.014 |
| ABL2 | 1983.9 ± 310.22 | -0.216 | 0.031 |
| kallikrein 12 | 3006.5 ± 326.33 | -0.208 | 0.038 |
| UNC5H4 | 6538.4 ± 1442.48 | 0.236 | 0.018 |
| RBM39 | 398.1 ± 51.47 | 0.208 | 0.039 |
| ERP29 | 4204.9 ± 909.60 | -0.206 | 0.040 |
| Galectin-2 | 999.2 ± 135.92 | 0.306 | 0.002 |
| CAMK1 | 7594.7 ± 994.07 | -0.204 | 0.042 |
| FGFR-2 | 356.3 ± 74.14 | 0.227 | 0.030 |
| IL-5 | 10041.2 ± 1820.08 | -0.233 | 0.019 |
| MIA | 3414.5 ± 842.41 | 0.204 | 0.042 |
| IL-7 Ra | 483.6 ± 60.95 | 0.235 | 0.022 |
| PBEF | 1545.2 ± 407.27 | 0.204 | 0.043 |
| Cytidylate kinase | 9979.7 ± 1689.67 | -0.248 | 0.013 |
| HCC-4 | 27279.2 ± 6606.88 | -0.200 | 0.046 |
| PPIB | 5161.1 ± 778.59 | -0.213 | 0.033 |
| HDAC8 | 1876.7 ± 238.46 | -0.200 | 0.047 |
| Fibrinogen g-chain dimer | 7544.1 ± 1696.32 | 0.218 | 0.031 |
| MASP3 | 5227.9 ± 798.27 | -0.262 | 0.009 |
| ADAM 9 | 2568.4 ± 417.47 | 0.200 | 0.048 |
| Protein disulfide isomerase A3 | 309.6 ± 27.93 | -0.225 | 0.024 |
| Calcineurin B a | 15197.6 ± 2217.91 | -0.199 | 0.048 |
| IGFBP-3 | 1126.8 ± 209.47 | 0.255 | 0.011 |
| PDE3A | 1341.0 ± 172.70 | -0.216 | 0.032 |
| MMP-8 | 949.7 ± 227.37 | 0.197 | 0.050 |
| Kininogen HMW | 34062.4 ± 9206.59 | -0.217 | 0.031 |

Results obtained from Pearson’s correlation analysis, with correlation coefficients and p ≤ 0.05 presented.
