# Supplementary material for: A proteomic signature that reflects pancreatic beta-cell function
Source: PLoS One. 2018 Aug 30;13(8):e0202727. doi: 10.1371/journal.pone.0202727 (PMC6117012; doi:10.1371/journal.pone.0202727)
Supplement: S6 Table — All values are means ± standard deviation. BMI, Body Mass Index; BP SYS, Systolic Blood Pressure; BP DIA, Diastolic Blood Pressure; HOMA-IR, Homeostatic Model Assessment of Insulin Resistance; BCF/HOMA IR, beta-cell function adjusted by HOMA IR. (DOCX) [file pone.0202727.s006.docx]

**S6 Table. Baseline characteristics FHI cohort (n=45)**

| Variable | Mean ± S.D. |
| --- | --- |
| Sex (m/f) | 26/19 |
| Age (y) | 53 ± 7 |
| Weight (kg) | 92.2 ± 15.9 |
| BMI (kg/m^2^) | 31.6 ± 4.8 |
| Waist (cm) | 93.8 ± 11.4 |
| BP SYS (mm/Hg) | 124.39 ± 13.94 |
| BP DIA (mm/Hg) | 81.34 ± 7.02 |
| Glucose (mmol/L) | 5.67 ± 0.66 |
| Insulin (µIU/mL) | 11.98 ± 9.99 |
| HOMA-IR | 3.12 ± 2.86 |
| Beta-cell function/HOMA-IR (pmol/mmol) | 13.41 ± 10.40 |
| Disposition Index (pmol/mmol) | 3.21 ± 2.32 |
| Calcineurin (ng/mL) | 0.63 ± 0.47 |

All values are means ± standard deviation. BMI, Body Mass Index; BP SYS, Systolic Blood Pressure; BP DIA, Diastolic Blood Pressure; HOMA-IR, Homeostatic Model Assessment of Insulin Resistance; BCF/HOMA IR, beta-cell function adjusted by HOMA IR
