# Supplementary material for: A proteomic signature that reflects pancreatic beta-cell function
Source: PLoS One. 2018 Aug 30;13(8):e0202727. doi: 10.1371/journal.pone.0202727 (PMC6117012; doi:10.1371/journal.pone.0202727)
Supplement: S1 Fig — Protein concentrations displayed in relative fluorescence units (RFU’s). (DOCX) [file pone.0202727.s007.docx]

**
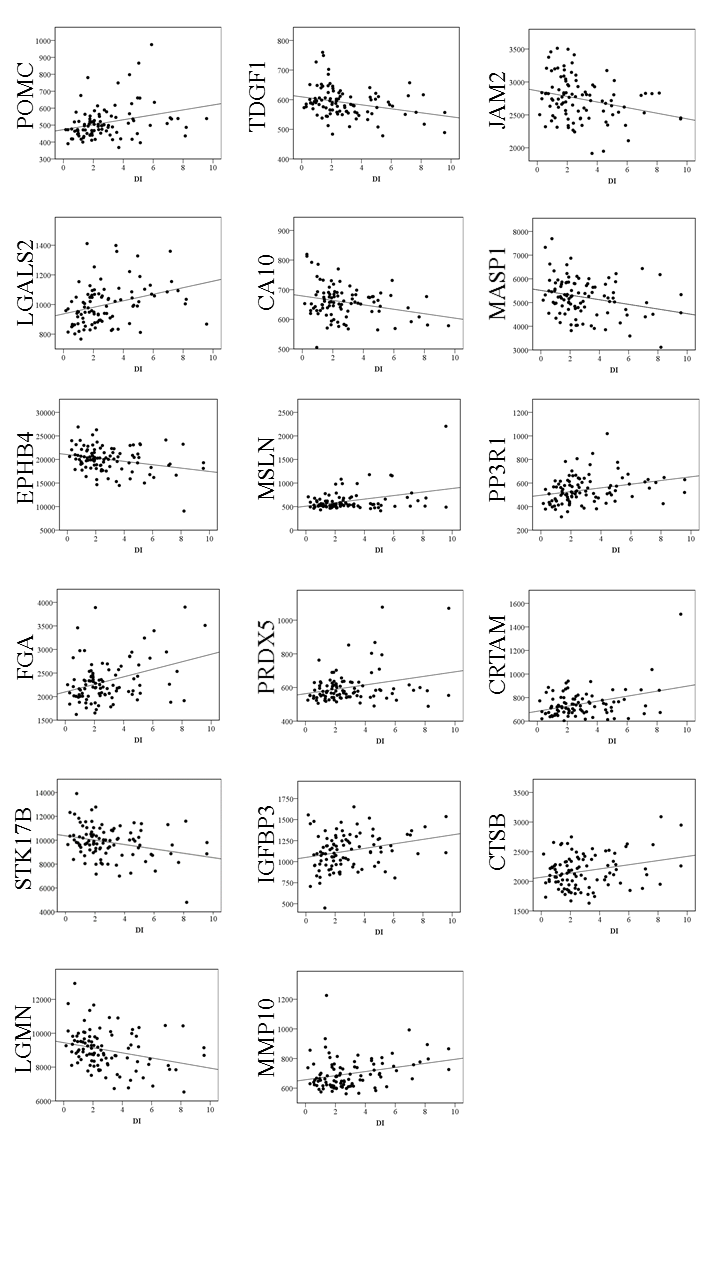
**

**S1 Fig. Scatterplots of proteins related to disposition index (DI) from Pearson’s correlation analysis (p ≤ 0.01).** Protein concentrations displayed in relative fluorescence units (RFU’s).
