# Supplementary material for: A proteomic signature that reflects pancreatic beta-cell function
Source: PLoS One. 2018 Aug 30;13(8):e0202727. doi: 10.1371/journal.pone.0202727 (PMC6117012; doi:10.1371/journal.pone.0202727)
Supplement: S2 Fig — Par scaling was used and random forest classification method was selected. Using all 21 variables, the best ROC curve was produced with an AUC of 0.918. AUC: area under the curve. A) Features ranked by mean importance measure for the receiver operating characteristic curve. Tertile 1: low beta-cell function Tertile 3: high beta-cell function Green filled square: Low concentration/value Red filled square: High concentration/value Green filled square/Red filled square: Positive association with beta-cell function/HOMA-IR Red filled square/Green filled square: Inverse association with beta-cell function/HOMA-IR B) Probabilities of predicted belonging to the low beta-cell function. Overall 26 participants were classified correctly into low beta-cell function (1) and high beta-cell function (3). Four participants were not correctly classified. C) An overview of the predictive accuracies using different amounts of variables. Using all 21 variables, the model is 83% accurate in predicting participants to have low or high beta-cell function. (DOCX) [file pone.0202727.s008.docx]

**
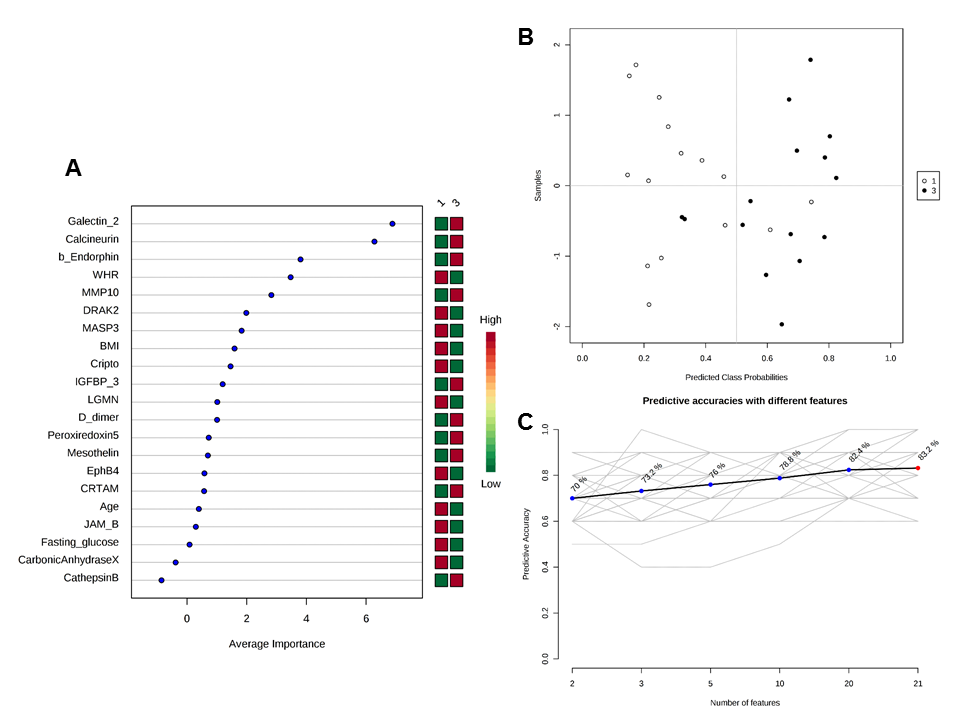
**

**S2 Fig.** **The receiver operating characteristic curve analysis to assess the predictive ability of ability of the protein panel (17 proteins associated with the disposition index (p ≤ 0.01)), age, BMI, waist to hip ratio and fasting glucose, for classification of low and high disposition index values of MECHE participants (n = 30).** Par scaling was used and random forest classification method was selected. Using all 21 variables, the best ROC curve was produced with an AUC of 0.918. AUC: area under the curve.

**A)** Features ranked by mean importance measure for the receiver operating characteristic curve. Tertile 1: low beta-cell function Tertile 3: high beta-cell function

Low concentration/value High concentration/value

Positive association with beta-cell function/HOMA-IR

Inverse association with beta-cell function/HOMA-IR

**B)** Probabilities of predicted belonging to the low beta-cell function. Overall 26 participants were classified correctly into low beta-cell function (1) and high beta-cell function (3). Four participants were not correctly classified.

**C)** An overview of the predictive accuracies using different amounts of variables. Using all 21 variables, the model is 83% accurate in predicting participants to have low or high beta-cell function.
