# Supplementary material for: A proteomic signature that reflects pancreatic beta-cell function
Source: PLoS One. 2018 Aug 30;13(8):e0202727. doi: 10.1371/journal.pone.0202727 (PMC6117012; doi:10.1371/journal.pone.0202727)
Supplement: S3 Fig — Par scaling was used and random forest classification method was selected. Using all 26 variables, the best ROC curve was produced with an AUC of 0.913. AUC: area under the curve. (DOCX) [file pone.0202727.s009.docx]

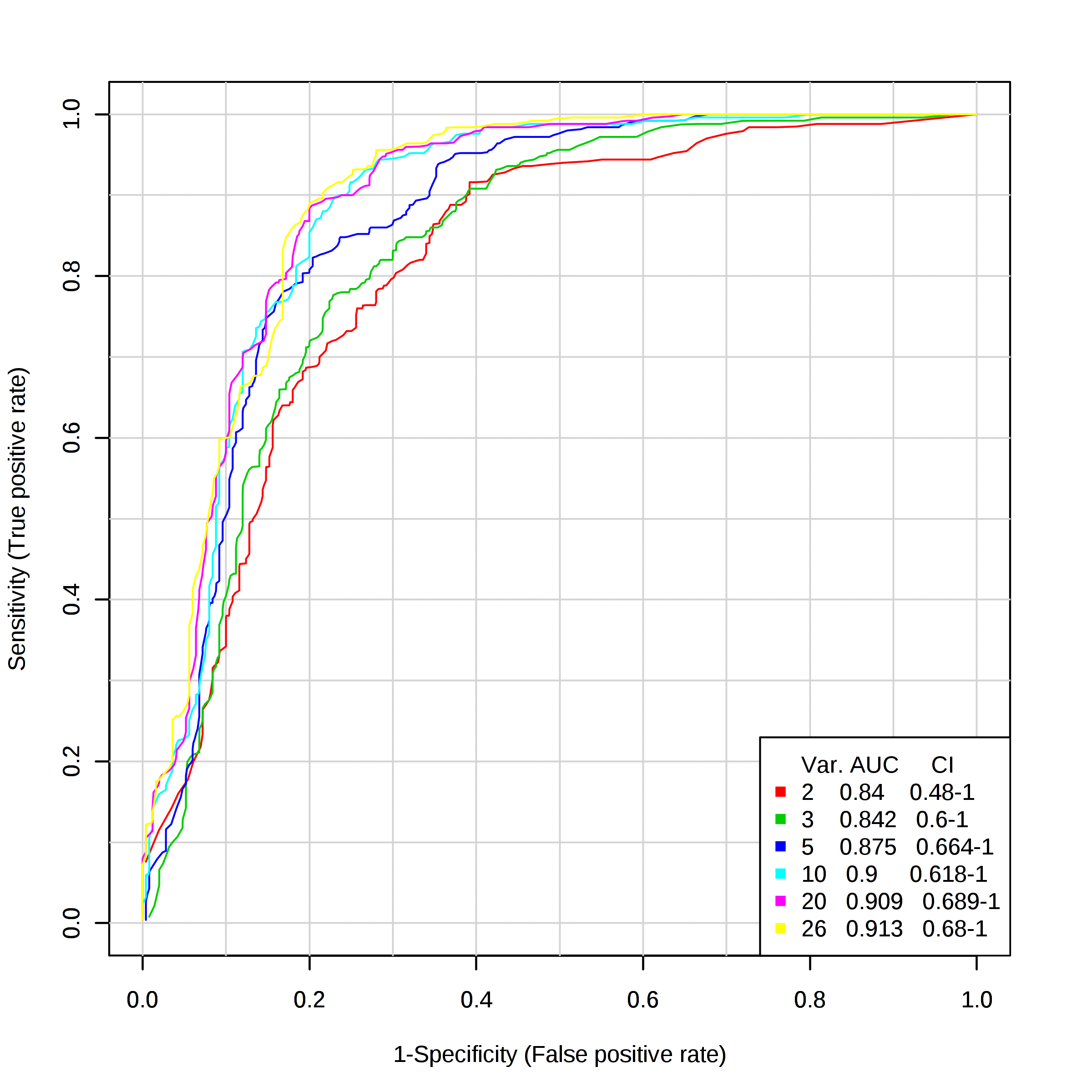


**S3 Fig.** **The receiver operating characteristic curve to assess the predictive ability of the protein panel (22 proteins associated with the beta-cell function/HOMA-IR (p ≤ 0.01)), age, BMI, waist to hip ratio and fasting glucose, for classification of low and high beta-cell function/ HOMA-IR values of MECHE participants (n=30).** Par scaling was used and random forest classification method was selected. Using all 26 variables, the best ROC curve was produced with an AUC of 0.913. AUC: area under the curve.
