# Supplementary material for: A proteomic signature that reflects pancreatic beta-cell function
Source: PLoS One. 2018 Aug 30;13(8):e0202727. doi: 10.1371/journal.pone.0202727 (PMC6117012; doi:10.1371/journal.pone.0202727)
Supplement: S4 Fig — Green filled square: Up-regulated with increasing beta-cell functionRed filled square: Down-regulated with increasing beta-cell functionYellow Filled square: Measured but no association (DOCX) [file pone.0202727.s010.docx]

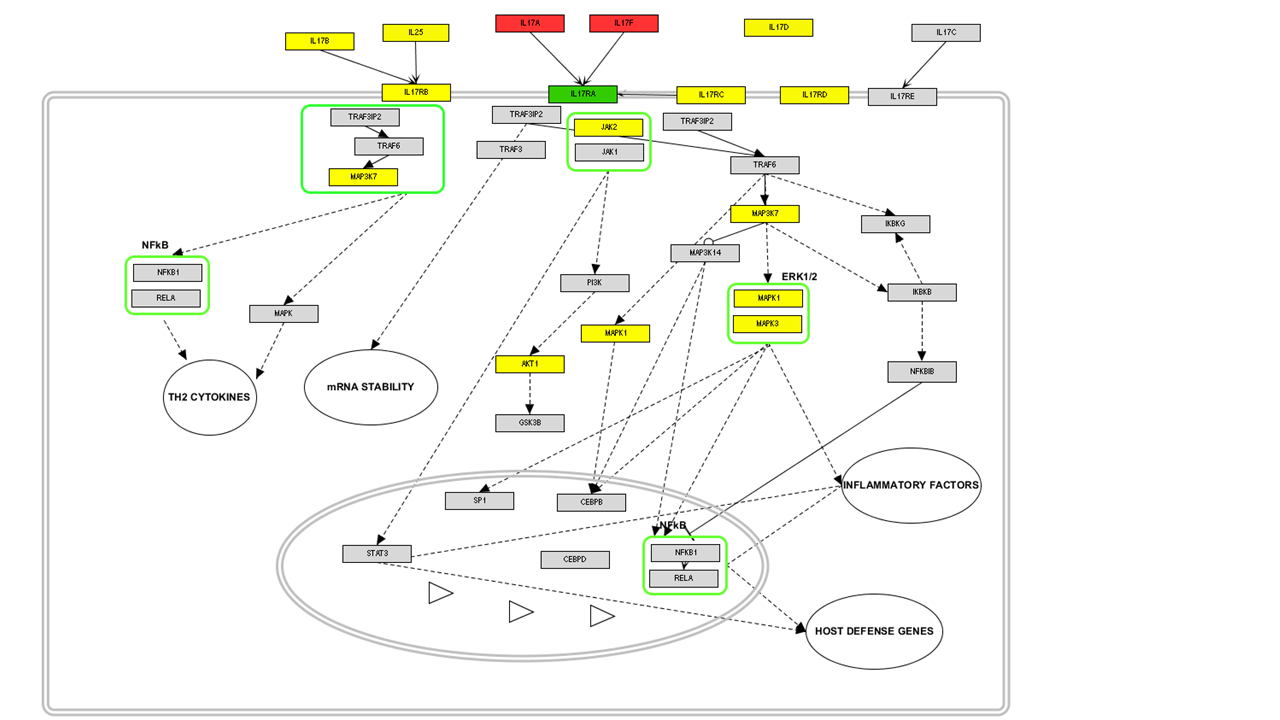


**S4 Fig. IL-17 signalling pathway obtained from WikiPathways displaying proteins significantly associated with beta-cell function measures.**

Up-regulated with increasing beta-cell function Down-regulated with increasing beta-cell function

Measured but no association
