# Supplementary material for: A proteomic signature that reflects pancreatic beta-cell function
Source: PLoS One. 2018 Aug 30;13(8):e0202727. doi: 10.1371/journal.pone.0202727 (PMC6117012; doi:10.1371/journal.pone.0202727)
Supplement: S5 Fig — Values are expressed as mean ± SD. No significant differences were observed. Sodium azide is the negative control. (DOCX) [file pone.0202727.s011.docx]

**S5 Fig. Cell viability of BRIN-BD11 cells following treatment with different concentrations of IL-17F for 20 h (n = 4).** Values are expressed as mean ± SD. No significant differences were observed. Sodium azide is the negative control.
